# Synthesis and Herbicidal Activity of 5-Heterocycloxy-3-methyl-1-substituted-1*H*-pyrazoles

**DOI:** 10.3390/molecules21010039

**Published:** 2015-12-25

**Authors:** Jing Kang, Xia Li Yue, Chang Shui Chen, Jian Hong Li, Hong Ju Ma

**Affiliations:** 1 Department of Applied Chemistry, College of Science, Huazhong Agricultural University, Wuhan 430070, China; kangjing@webmail.hzau.edu.cn (J.K.); yxl@mail.hzau.edu.cn (X.L.Y.); chenchang@mail.hzau.edu.cn (C.S.C.); 2 Department of Plant Protection, College of Plant Science and Technology, Huazhong Agricultural University, Wuhan 430070, China; jianhl@mail.hzau.edu.cn

**Keywords:** pyrazole, synthesis, herbicidal activities

## Abstract

With the objective of finding valuable herbicidal candidates, a series of new 5-heterocycloxy-3-methyl-1-substituted-1*H*-pyrazoles were synthesized and their herbicidal activities were evaluated. The bioassay results showed that some compounds exhibited excellent herbicidal activities at the concentration of 100 mg/L, and compound 5-chloro-2-((3-methyl-1-(2,2,2-trifluoroethyl)-1*H*-pyrazol-5-yl)oxy)pyrimidine showed bleaching activity to green weeds. In greenhouse conditions, this compound also showed excellent post-emergence herbicidal effect against *Digitaria sanguinalis* L. at the dosage of 750 g a. i. ha^−1^.

## 1. Introduction

**Figure 1:**
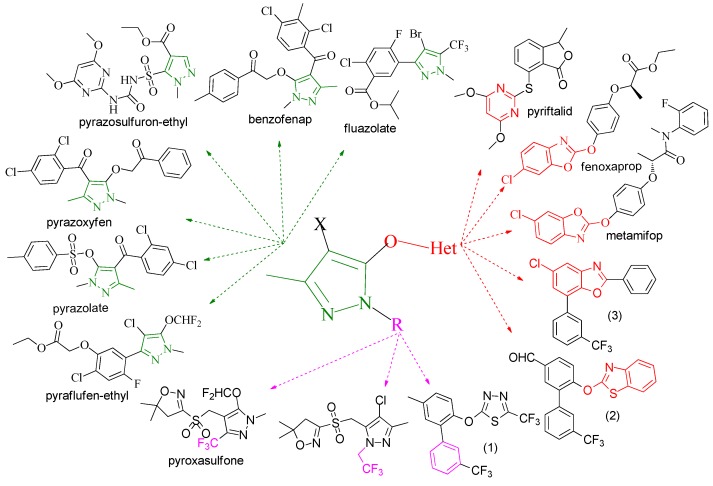
Structural importance of few of the molecules.

Pyrazole derivatives not only occupy an important position in medicinal chemistry due to their wide range of bioactivities such as anticancer [1], analgesic [2], anti-convulsant [3], anti-depressant [4], anti-inflammatory [5], antibacterial [6] antimalarial [6], and antituberculosis activity [6], but also has been drawn great attention in agrochemicals because of their excellent bioactivity such as the commercialized herbicides pyrazolate, pyrazoxyfen, benzofenap, pyraflufen-ethyl, fluazolate, and pyrazosulfuron-ethyl [7,8,9,10,11,12,13,14,15]. Owing to the interesting applications of pyrazoles in the field of agricultural research, the combination of such pyrazole molecule with the additional heterocycles to form polycyclic systems to add functional diversity, is increasingly becoming a fruitful area of the study for their biological activity [16]. Compounds with fused heterocycles showed excellent bioactivity, such as metamifop, fenoxaprop, and pyriftalid [17,18]. Plants treated with herbicides inhibiting carotenoid biosynthesis show characteristic white foliage. Carotenoids protect chlorophyll from photooxidation and chlorophyll is destroyed as it is formed in tissues being devoid of carotenoids. One of the well-studied sites of carotenoid biosynthesis inhibition is that of phytoene desaturase (PDS) and many reviews have been published [19,20,21,22,23,24,25,26]. Despite the great number of structurally diverse inhibitors of phytoene desaturase have been known, the enzyme is still a good target site for new herbicides owing to their good selective toxicity. This has led to the discovery of 2-((5-methyl-3’-(trifluoromethyl)-[1,1’-biphenyl]-2-yl)oxy)-5-(trifluoromethyl)-1,3,4-thiadiazole (1) or 6-(benzothiazol-2-yloxy)-3’-(trifluoromethyl)-[1,1’-biphenyl]-3-carbaldehyde (2) and 5-chloro-2-phenyl-7-(3-(trifluoromethyl)phenyl)benzoxazole (3) as bleaching herbicides candidates [23,27]. The introduction of trifluoromethyl into *N*-methyl group of pyrazole ring would be expected to improve herbicidal activity due to the intrinsic properties of trifluoromethyl, such as high thermal stability, increased lipophilicity, its electronegativity, and relatively small size [28,29]. The chemical structures of the compounds mentioned above was represented in Figure 1. The pyrazolylpyrimidine derivatives have been reported to show inhibition of chlorophyll and carotenoid biosynthesis in our previous work [30,31]. In view of the above mentioned facts and in continuation of our interest in the synthesis of pyrazole heterocycles, the synthesis and herbicidal activities of these novel pyrazole derivatives are described in this paper.

## 2. Results and Discussion

### 2.1. Synthesis

**Scheme 1:**
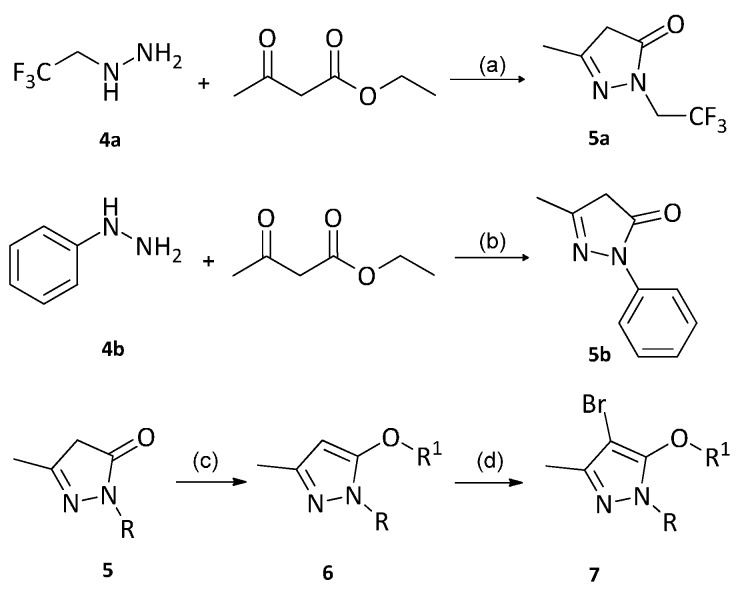
Synthetic route of target compounds. *Reagents and conditions*: (a) C_2_H_5_OH, reflux, 3 h; (b) C_2_H_5_OH/H_2_O = 1:2, HCl, rt, 5 min, 60 °C, 1.5 h, pH = 7; (c) R^1^-Cl, DMSO, K_2_CO_3_, 3 h; (d) NBS, DMF, rt, overnight.

The synthetic route of a series of pyrazole derivatives were shown in Scheme 1. The chemical structures of fourteen target compounds were listed in Table 1. The structures of these compounds were confirmed by ^1^H-NMR (see Supplementary Materials), ^13^C-NMR (see Supplementary Materials), MS, Elemental analysis, and FT-IR. The compound **5b** was synthesized according to the method of preparing 3,5-dimethyl-1-(2,2,2-trifluoroethyl)-1-*H*-pyrazole reported in the literature [29].

**Table 1:** Chemical structure of target compounds

Compd.	R	R^1^	Compd.	R	R^1^
**6a**	CH_2_CF_3_	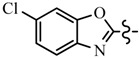	**6h**	C_6_H_5_	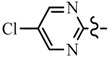
**6b**	CH_2_CF_3_	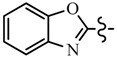	**6i**	C_6_H_5_	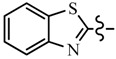
**6c**	CH_2_CF_3_	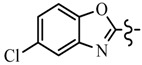	**7a**	CH_2_CF_3_	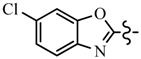
**6d**	CH_2_CF_3_	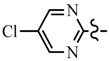	**7b**	CH_2_CF_3_	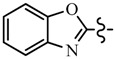
**6e**	C_6_H_5_	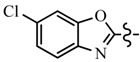	**7c**	CH_2_CF_3_	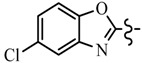
**6f**	C_6_H_5_	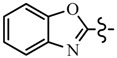	**7d**	CH_2_CF_3_	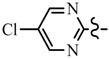
**6g**	C_6_H_5_	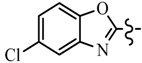	**7e**	C_6_H_5_	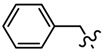

### 2.2. Growth Inhibition of Weed Roots and Shoots

The herbicidal activities of the target compounds were determined with *Brassica campestris* L. (*B. campestris*), *Amaranthus retroflexus* L. (*A. retroflexus*) and *Portulaca oleracea* L. (*P. oleracea*) as samples of annual dicotyledonous plants and *Pennisetum alopecuroides* L. (*P. alopecuroides*), *Echinochloa crus-galli* L. (*E. crus-galli*), and *Digitaria sanguinalis* L. (*D. sanguinalis*) as samples of annual monocotyledonous plants. The results of the inhibition effect were shown in Table 2. All compounds showed inhibitory effect on the roots growth of *B. campestris*, *A. retroflexus*, *P. oleracea*, and *D. sanguinalis* and the shoots growth of *P. alopecuroides* and *E. crus-galli*. Compounds **6d** and **7d** showed excellent inhibition effect against all tested dicotyledonous and monocotyledonous plants at 100 mg/L. Compound **6d** exhibited excellent bleaching activities to *B. campestris*, *P. alopecuroides*, *E. crus-galli*, and *D. sanguinalis*, and even bleached *D. sanguinalis* at the concentration of 10 mg/L. Among these target compounds, only compound **6d** showed best bleaching activity. It was possible that pyrimidine group played a key role in bleaching phytotoxicity. Comparing the compounds **6d** and **7d**, the presence of bromine at the 4-position on the pyrazole ring had a negative effect on bleaching activity.

It could be seen from Table 2, compounds **6a**–**6d** showed better herbicidal effect than compounds **6e**–**6i**. The derivatives with 2,2,2-trifluoroethyl at the 1-position on pyrazole showed better inhibition effect than that of compounds substituted with the phenyl group, indicating the 2,2,2-trifluoroethyl group on the pyrazole ring played an important role in inhibition. Compounds **6d** and **6h** had better inhibition activities than compounds **6a**–**6c** and **6e**–**6g**, indicating that the bulkiness of fused heterocyclic substitute at pyrazole ring might be attributable to the decreased of herbicidal activity. Comparing the activities of compounds **6** and compounds **7**, the electron-withdrawing bromine group at C-4 had no beneficial effect on activity.

**Table 2:** Inhibition of target compounds on the growth of weed.

Compd.	Relative Inhibition (%)											
	*B. campestris*		*A. retroflexus*		*P. oleracea*		*P. alopecuroides*		*E. crus-galli*		*D. sanguinalis*	
	Root		Root		Root		Shoot		Shoot		Root	
	10 mg/L	100 mg/L	10 mg/L	100 mg/L	10 mg/L	100 mg/L	10 mg/L	100 mg/L	10 mg/L	100 mg/L	10 mg/L	100 mg/L
**6a**	69 ± 2.1	78 ± 1.1	39 ± 1.1	58 ± 2.1	3 ± 1.3	48 ± 1.6	47 ± 1.0	59 ± 0.5	47 ± 1.1	57 ± 2.3	27 ± 1.5	58 ± 1.1
**6b**	80 ± 1.2	81 ± 1.1	53 ± 1.7	60 ± 2.4	29 ± 1.2	50 ± 0.8	56 ± 2.2	59 ± 1.6	60 ± 0.6	70 ± 1.3	31 ± 2.9	75 ± 0.7
**6c**	37 ± 1.5	46 ± 2.0	26 ± 3.3	58 ± 1.5	30 ± 1.7	74 ± 2.1	38 ± 2.7	55 ± 1.0	26 ± 1.4	61 ± 0.6	9 ± 2.4	21 ± 0.8
**6d**	49 ± 2.4	W	41 ± 0.3	100	42 ± 1.8	100	34 ± 1.6	W	30 ± 2.0	W	W	W
**6e**	21 ± 0.5	22 ± 1.6	0 ± 1.2	27 ± 2.5	4 ± 2.2	32 ± 1.9	22 ± 1.2	32 ± 1.2	33 ± 2.4	36 ± 2.7	2 ± 1.4	28 ± 0.7
**6f**	42 ± 1.3	54 ± 2.1	0 ± 1.3	7 ± 1.7	3 ± 2.0	23 ± 2.0	30 ± 1.9	58 ± 1.9	30 ± 2.4	52 ± 0.5	36 ± 1.3	46 ± 1.3
**6g**	16 ± 1.0	36 ± 1.0	3 ± 1.8	13 ± 0.3	11 ± 0.5	53 ± 0.7	21 ± 1.4	34 ± 1.1	12 ± 1.6	24 ± 1.5	2 ± 4.4	7 ± 3.5
**6h**	17 ± 1.6	62 ± 3.0	9 ± 0.2	35 ± 1.5	41 ± 1.0	66 ± 0.7	40 ± 1.9	63 ± 1.6	29 ± 1.5	53 ± 0.8	1 ± 1.5	W
**6i**	61 ± 1.4	64 ± 0.5	6 ± 1.1	29 ± 1.2	3 ± 2.6	32 ± 0.9	48 ± 1.3	54 ± 1.7	31 ± 1.4	37 ± 2.1	9 ± 2.8	22 ± 2.2
**7a**	27 ± 1.4	59 ± 2.4	25 ± 2.2	53 ± 0.6	11 ± 2.2	55 ± 0.5	24 ± 2.8	41 ± 0.8	27 ± 2.3	54 ± 2.3	32 ± 2.4	74 ± 0.3
**7b**	36 ± 1.7	53 ± 0.9	39 ± 1.0	58 ± 1.0	34 ± 0.7	61 ± 0.4	48 ± 1.7	78 ± 1.7	53 ± 1.7	78 ± 1.7	44 ± 1.9	67 ± 1.9
**7c**	22 ± 1.2	72 ± 0.5	8 ± 3.5	20 ± 2.1	25 ± 1.8	49 ± 2.0	37 ± 2.1	56 ± 1.4	18 ± 0.1	68 ± 0.2	28 ± 2.1	63 ± 0.2
**7d**	46 ± 1.6	79± 0.5	46 ± 1.8	79 ± 1.0	48 ± 2.1	72 ± 0.8	49 ± 2.2	77 ± 0.6	22 ± 0.6	83 ± 1.1	45 ± 1.5	77 ± 0.7
**7e**	32 ± 3.5	64 ± 1.7	4 ± 0.7	15 ± 0.7	3 ± 1.3	23 ± 1.4	32 ± 1.5	61 ± 0.4	21 ± 1.7	43 ± 2.5	13 ± 1.4	22 ± 2.5

W: Leaves were completely white after treatment; *B. campestris*, *Brassica campestris* L.; *A. retroflexus*, *Amaranthus retroflexus* L.; *P. oleracea*, *Portulaca oleracea* L.; *P. alopecuroides*, *Pennisetum alopecuroides* L.; *E. crus-galli*, *Echinochloa crus-galli* L.; *D. sanguinalis*, *Digitaria sanguinalis* L.

### 2.3. Screening in Greenhouse Conditions

Seven target compounds **6a**–**6d**, **7a**, **7b**, **7d** with higher inhibitory effects on the growth of tested plants in preliminary herbicidal bioassays were further screened in greenhouse conditions. From the biological assay results in Table 3, each compound showed herbicidal activities in postemergence treatment at the dosage of 750 g a. i. ha^−1^, especially monocotyledonous weed *D. sanguinalis* was most sensitive to compound **6d***.* It was also found that when the 5-position of the pyrazole was modified by a pyrimidine group, compound **6d** had better inhibitory effect on *D. sanguinalis* than other target compounds, the inhibition rate of the fresh weights reaching 82%. The bioassay results indicated that the substituted group at the 5-position of the pyrazole ring played an important role for herbicidal activity. The 4-[5-methyl-3-(trifluoromethyl)-1*H*-pyrazol-1-yl]-6-(prop-2-yn-1-yloxy)pyrimidine reported in our previous work [30] was very close to compound **6d**, structurally, but it showed less herbicidal activity than compound **6d** in greenhouse conditions, the difference between these two structures lay in that the pyrimidine was substituted at the 1-position of the pyrazole ring [30] and at the 5-position of the pyrazole ring in this paper, we might conclude that the substituted position on the pyrazole ring also played an important role for herbicidal activity.

**Table 3:** Herbicidal activities of compounds in greenhouse conditions

Compd.	Relative Inhibition (%)					
	*A. theophrasti*	*A. retroflexus*	*P. oleracea*	*P. alopecuroides*	*E. crus-galli*	*D. sanguinalis*
**6a**	22 ± 1.3	27 ± 1.4	30 ± 1.2	42 ± 1.4	34 ± 2.6	21 ± 2.7
**6b**	35 ± 2.0	29 ± 0.7	40 ± 1.1	25 ± 0.8	26 ± 2.2	17 ± 2.0
**6c**	40 ± 1.0	44 ± 0.3	38 ± 1.7	32 ± 1.6	30 ± 1.6	33 ± 2.6
**6d**	42 ± 2.0	50 ± 0.9	30 ± 0.7	6 ± 2.7	41 ± 2.6	82 ± 0.9
**7a**	21 ± 0.6	46 ± 1.6	34 ± 2.6	12 ± 3.1	60 ± 0.5	56 ± 0.6
**7b**	9 ± 0.6	56 ± 0.9	34 ± 1.5	5 ± 2.1	50 ± 2.3	46 ± 1.7
**7d**	11 ± 2.2	37 ± 1.8	28 ± 1.3	17 ± 2.7	48 ± 0.4	52 ± 0.3

*A. theophrasti*, *Abutilon theophrasti* M.; *A. retroflexus*, *Amaranthus retroflexus* L.; *P. oleracea*, *Portulaca oleracea* L.; *P. alopecuroides*, *Pennisetum alopecuroides* L.; *E. crus-galli*, *Echinochloa crus-galli* L.; *D. sanguinalis*, *Digitaria sanguinalis* L.

### 2.4. Inhibitory Effect of the Compound ***6d*** on Chlorophyll of Weed

In order to evaluate the bleaching activities of compound **6d**, the changes in chlorophyll contents of treated seedlings were tested. The IC_50_ value of compound **6d** and positive control (diflufenican) were shown in Table 4. Compound **6d** inhibited the synthesis of chlorophyll, and showed the same inhibition activity as commercial standard diflufenican against *B. campestris.* Gramineous weed *P. alopecuroides*, *E. crus-galli*, and *D. sanguinalis* were slightly sensitive to diflufenican than compound **6d** and compound **6d** deserved further studies on structure optimization and biological efficacy as the bleaching herbicidal inhibitor.

**Table 4:** Determination of chlorophyll inhibition of compound **6d.**

Species	6d		Diflufenican	
	IC_50_ (95% Confidence Intervals) (mg·L^−1^)	Slope (±SE)	IC_50_ (95% Confidence Intervals) (mg·L^−1^)	Slope (±SE)
*B. campestris*	20.01 (16.48–24.28)	3.54 ± 0.32	19.79 (11.23–34.90)	1.15 ± 0.39
*P. alopecuroides*	11.71 (10.57–12.98)	1.53 ± 0.08	5.97 (4.04–8.82)	0.45 ± 0.06
*E. crus-galli*	6.14 (5.83–6.46)	2.09 ± 0.04	0.88 (0.07–9.81)	2.24 ± 0.28
*D. sanguinalis*	8.09 (6.08–10.76)	4.17 ± 0.34	1.09 (0.36–3.24)	3.12 ± 0.16

*B. campestris*, *Brassica campestris* L.; *P. alopecuroides*, *Pennisetum alopecuroides* L.; *E. crus-galli*, *Echinochloa crus-galli* L.; *D. sanguinalis*, *Digitaria sanguinalis* L.

## 3. Experimental Section

### 3.1. Analysis and Instruments

Elemental analyses (C, H, N) were performed with a Vario EL III elemental analyzer (Elementar Analysensysteme Gmbh, Hanau, Germany) at the Institute of Chemistry, Chinese Academy of Sciences. Infrared spectra were taken on a Nicolet IR 200 FT-IR instrument (Thermo Scientific, Waltham, MA, USA). ^1^H-NMR spectra and ^13^C-NMR were obtained at 400 MHz using a Bruker AVANCE III 400 spectrometer (Bruker Corp., Billerica, MA, USA) in CDCl_3_ solution with TMS as an internal standard. Mass spectra (MS) were recorded in a Agilent 6110 spectrometer (Agilent, Santa Clara, CA, USA). Melting points were measured on a SGW X-4 melting point apparatus (Shanghai Precision Scientific Instrument Co., Ltd., Shanghai, China) and are uncorrected. All reagents and solvents were obtained from commercial suppliers.

### 3.2. Synthesis and Characterization of Target Compounds

#### 3.2.1. Synthesis of Compounds **5a** and **5b**

A solution of 2,2,2-trifluoroethylhydrazine (0.03 mol, 70% in water) and ethyl acetoacetate (0.03 mol) in ethanol (50 mL) was stirred at 60 °C for 3 h. The solvent was removed *in vacuo*, and the resulting residue was purified by silica gel column chromatography (ethyl acetate/petroleum ether: 1/4 as the eluent) to give the white solid 3-methyl-1-(2,2,2-trifluoromethyl)-1*H*-pyrazol-3-one (**5a**). Yield 60.2%; m.p. 105–106 °C; ^1^H-NMR (CDCl_3_, 400 MHz), δ (ppm): 2.13 (s, 3H, CH_3_), 3.25 (s, 2H, CH_2_), 4.19–4.25 (q, 2H, *J* = 8.8 Hz, CH_2_CF_3_,); ESI-MS [M + H]^+^: 181.1.

To a solution of ethyl acetoacetate (0.03 mol) in water (12 mL) and ethanol (6 mL) phenylhydrazine (0.03 mol) was added. After stirring for 2 min at room temperature, 1 mL of 36.5% concentrated hydrochloric acid was added and stirred at room temperature for 5 min, then at 60 °C for 1.5 h. The reaction mixture was treated with 10% sodium hydroxide solution to pH 7, after stirring for 10 min, the precipitate was filtered, washed with distilled water, and dried to give a light yellow solid as 3-methyl-1-phenyl-2-pyrazoline-5-one (**5b**). Yield 80.5%; m.p. 126–127 °C; ^1^H-NMR (CDCl_3_, 400 MHz), δ (ppm): 2.27 (s, 3H, CH_3_), 3.43 (s, 2H, pyrazole-4H), 7.18–7.23 (m, 1H, ArH,), 7.37–7.43 (m, 2H, ArH), 7.84–7.87 (t, 2H, *J*= 8.8 Hz, ArH); ESI-MS [M + H]^+^: 175. The compound **5b** was a known compound and its experimental data in the literature [32,33] was shown as follows: m.p., 126–128 °C, ^1^H-NMR (400 MHz, CDCl_3_), δ (ppm): 2.13 (s, 3H, CH_3_), 3.37 (s, 2H, pyrazole-4H), 7.13 (t, *J* = 7.6 Hz, 1H, ArH), 7.35 (t, *J* = 8.0 Hz, 2H, ArH), 7.81 (d, *J* = 7.6 Hz, 2H, ArH).

#### 3.2.2. Synthesis of Compounds **6a**–**6i**

Compound **5a** (1.5 mmol) and powdered potassium carbonate (2.25 mmol) were added into dimethylsulfoxide (20 mL). 2, 6-Dichlorobenzoxazole (1.5 mmol) was added to the solution and the mixture was stirred at room temperature for 3 h. The mixture was diluted with water (20 mL) and extracted with diethyl ether (30 mL × 2). The extract was washed with water and saturated brine, dried over anhydrous sodium sulfate, the filtrate was concentrated *in vacuo*. The residue was further purified by silica gel column chromatography (ethyl acetate/petroleum ether: 1/10 as the eluent) to give compound **6a** as a white solid. Compounds **6b**–**i** were synthesized using the same procedures.

*6-Chloro-2-((1-(2,2,2-trifluoroethyl)-3-methyl-1H-pyrazol-5-yl)oxy)benzoxazole* (**6a**), white solid, yield 69.6%, m.p. 105–106 °C; IR (KBr, ν_max_, cm^−1^): 2919 (CH_3_), 1637 (-C=N-), 1355 (C-F), 1260 (=C-O-C), 1164 (-C-O-C). ^1^H-NMR (CDCl_3_, 400 MHz), δ (ppm): 2.24 (s, 3H, CH_3_), 4.55–4.62 (q, 2H, *J* = 8.24 Hz, CH_2_CF_3_), 6.33 (s, 1H, pyrazole-4H), 7.25–7.27 (dd, 1H, *J* = 8.5, 1.8 Hz, ArH), 7.42–7.43 (d, 1H, *J* = 1.8 Hz, ArH), 7.43–7.45 (d, 1H, *J* = 8.6 Hz, ArH); ^13^C-NMR (CDCl_3,_ 100 MHz), δ (ppm): 13.57, 47.60 (q, ^2^*J*_C,F_ = 36 Hz), 92.38, 110.04, 118.87, 121.65 (q, ^1^*J*_C,F_ = 278 Hz), 124.69, 128.97, 137.74, 145.39, 147.39, 148.73, 157.90; ESI-MS [M + H]^+^: 332.1. Anal. Calcd for C_13_H_9_ClF_3_N_3_O_2_: C, 47.08; H, 2.74; N, 12.67. Found: C, 47.11; H, 2.72; N, 12.68.

*2-((1-(2,2,2-Trifluoroethyl)-3-methyl-1H-pyrazol-5-yl)oxy)benzoxazole* (**6b**), white solid, yield 69.7%, m.p. 83–84 °C; IR (KBr, ν_max_, cm^−1^): 2969 (CH_3_), 1629 (-C=N-), 1318 (C-F), 1257 (=C-O-C), 1175 (-C-O-C). ^1^H-NMR (CDCl_3_, 400 MHz), δ (ppm): 2.31 (s, 3H, CH_3_), 4.63–4.69 (q, 2H, *J* = 8.24 Hz, CH_2_CF_3_), 6.40 (s, 1H, pyrazole-4H), 7.29–7.36 (m, 2H, ArH), 7.45–7.48 (m, 1H, ArH), 7.59–7.61 (m, 1H, ArH); ^13^C-NMR (CDCl_3,_ 100 MHz), δ (ppm): 13.57, 47.60 (q, ^2^*J*_C,F_ = 36 Hz), 92.39, 110.04, 118.87, 121.65 (q, ^1^*J*_C,F_ = 278 Hz), 124.69, 128.99, 137.75, 145.40, 147.40, 148.73, 157.90; ESI-MS [M + H]^+^: 298.2. Anal. Calcd for C_13_H_10_F_3_N_3_O_2_: C, 52.53; H, 3.39; N, 14.14; Found: C, 52.50; H, 3.36; N, 14.13.

*5-Chloro-2-((1-(2,2,2-trifluoroethyl)-3-methyl-1H-pyrazol-5-yl)oxy)benzoxazole* (**6c**), white solid, yield 71.2%, m.p. 85–86 °C; IR (KBr, ν_max_, cm^−1^): 2924 (CH_3_), 1623 (-C=N-), 1307 (C-F), 1254 (=C-O-C), 1149 (-C-O-C). ^1^H-NMR (CDCl_3,_ 400 MHz), δ (ppm): 2.33 (s, 3H, CH_3_), 4.65–4.71 (q, 2H, *J* = 8.24 Hz, CH_2_CF_3_), 6.42 (s, 1H, pyrazole-4H), 7.30–7.33 (dd, 1H, *J* = 8.68, 2.12 Hz, ArH), 7.40–7.42 (d, 1H, *J* = 8.64 Hz, ArH); 7.60–7.61 (d, 1H, *J* = 2.0 Hz, ArH); ^13^C-NMR (CDCl_3,_ 100 MHz), δ (ppm): 14.59, 47.60 (q, ^2^*J*_C,F_ = 36 Hz), 93.44, 111.05, 119.51, 122.88 (q, ^1^*J*_C,F_ = 278 Hz), 124.72, 130.61, 141.14, 146.35, 146.92, 149.75, 159.40; ESI-MS [M + H]^+^: 332.1. Anal. Calcd for C_13_H_9_ClF_3_N_3_O_2_: C, 47.08; H, 2.74; N, 12.67; Found: C, 47.12; H, 2.72; N, 12.68.

*5-Chloro-2-((1-(2,2,2-trifluoroethyl)-3-methyl-1H-pyrazol-5-yl)oxy)pyrimidine* (**6d**), yellow solid, yield 47.9%, m.p. 56–57 °C; IR (KBr, ν_max_, cm^−1^): 2968 (CH_3_), 1626 (-C=N-), 1307 (C-F), 1259 (=C-O-C). ^1^H-NMR (CDCl_3_, 400 MHz), δ (ppm): 2.31 (s, 3H, CH_3_), 4.60–4.67 (q, 2H, *J* = 8.36 Hz, CH_2_CF_3_), 6.07 (s, 1H, pyrazole-4H), 8.59 (s, 2H, pyrimidine-4, 6H); ^13^C-NMR (CDCl_3_, 100 MHz), δ (ppm): 13.57, 47.42 (q, ^2^*J*_C,F_ = 36 Hz), 93.23, 121.77 (q, ^1^*J*_C,F_ = 278 Hz), 126.24, 146.36, 148.47, 157.16, 159.74; ESI-MS [M + H]^+^: 293.1. Anal. Calcd for C_10_H_8_ClF_3_N_4_O: C, 41.04; H, 2.76; N, 19.14; Found: C, 41.08; H, 2.75; N, 19.17.

*6-Chloro-2-((3-methyl-1-phenyl-1H-pyrazol-5-yl)oxy)benzoxazole* (**6e**), white solid, yield 64.5%, m.p. 78–79 °C; IR (KBr, ν_max_, cm^−1^): 2928 (CH_3_), 1634 (-C=N-), 1258 (=C-O-C), 1147 (-C-O-C). ^1^H-NMR (CDCl_3_, 400 MHz), δ (ppm): 2.40 (s, 3H, CH_3_), 6.46 (s, 1H, pyrazole-4H), 7.31–7.37 (m, 2H, ArH), 7.45–7.52 (m, 4H, ArH), 7.63–7.65 (d, 2H, *J* = 7.6 Hz, ArH); ^13^C-NMR (CDCl_3,_ 100 MHz), δ (ppm): 14.66, 94.53, 110.97, 119.80, 123.06, 125.57, 127.50, 129.22, 129.74, 137.64, 138.90, 145.08, 148.46, 149.11, 159.66; ESI-MS [M + H]^+^: 326.1. Anal. Calcd for C_17_H_12_ClN_3_O_2_: C, 62.68; H, 3.71; N, 12.90; Found: C, 62.75; H, 3.69; N, 12.92.

*2-((3-Methyl-1-phenyl-1H-pyrazol-5-yl)oxy)benzoxazole* (**6f**), white solid, yield 58.9%, m.p. 42–43 °C; IR (KBr, ν_max_, cm^−1^): 2927 (CH_3_), 1631 (-C=N-), 1256 (=C-O-C), 1153 (-C-O-C). ^1^H-NMR (CDCl_3,_ 400 MHz), δ (ppm): 2.40 (s, 3H, CH_3_), 6.47 (s, 1H, pyrazole-4H), 7.29–7.36 (m, 3H, ArH), 7.44–7.49 (m, 3H, ArH), 7.59–7.61 (dd, 1H, *J* = 8.88, 1.64 Hz, ArH), 7.65–7.66 (d, 2H, *J* = 7.72 Hz, ArH); ^13^C-NMR (CDCl_3_, 100 MHz), δ (ppm): 14.66, 94.54, 110.18, 119.26, 123.05, 124.21, 124.92, 127.40, 129.18, 137.63, 140.21, 145.34, 148.51, 149.10, 159.45; ESI-MS [M + H]^+^: 292.2. Anal. Calcd for C_17_H_13_N_3_O_2_: C, 70.09; H, 4.50; N, 14.42; Found: C, 70.05; H, 4.46; N, 14.42.

*5-Chloro-2-((3-methyl-1-phenyl-1H-pyrazol-5-yl)oxy)benzoxazole* (**6g**), white solid, yield 49.3%, m.p. 82–83 °C; IR (KBr, ν_max_, cm^−1^): 2924 (CH_3_), 1630 (-C=N-), 1254 (=C-O-C), 1174 (-C-O-C). ^1^H-NMR (CDCl_3_, 400 MHz), δ (ppm): 2.40 (s, 3H, CH_3_), 6.46 (s, 1H, pyrazole-4H), 7.26–7.28 (m, 1H, ArH), 7.33–7.38 (m, 2H, ArH), 7.45–7.49 (t, 2H, *J* = 7.34 Hz, ArH), 7.58 (d, 1H, *J* = 1.72 Hz, ArH), 7.63–7.65 (d, 2H, *J* = 7.88 Hz, ArH); ^13^C-NMR (CDCl_3_, 100 MHz), δ (ppm): 14.68, 94.91, 121.47, 122.26, 122.90, 124.79, 126.59, 127.23, 129.15, 132.37, 137.80, 147.36, 148.48, 149.03, 168.84. ESI-MS [M + H]^+^: 326.1. Anal. Calcd for C_17_H_12_ClN_3_O_2_: C, 62.68; H, 3.71; N, 12.90; Found: C, 62.75; H, 3.69; N, 12.92.

*5-Chloro-2-((3-methyl-1-phenyl-1H-pyrazol-5-yl)oxy)pyrimidine* (**6h**), yellow solid, yield 65.4%, m.p. 49–50 °C; IR (KBr, ν_max_, cm^−1^): 2951 (CH_3_), 1560 (-C=N-), 1290 (=C-O-C), 758 (C-Cl). ^1^H-NMR (CDCl_3_, 400 MHz), δ (ppm): 2.39 (s, 3H, CH_3_), 6.08 (s, 1H, pyrazole-4H), 7.24–7.28 (t, 1H, *J* = 7.48 Hz, ArH), 7.36–7.40 (t, 2H, *J* = 7.64 Hz, ArH), 7.62–7.64 (d, 2H, *J* = 7.68 Hz, ArH), 8.48 (s, 2H, pyrimidine-H); ^13^C-NMR (CDCl_3_, 100 MHz), δ (ppm): 14.65, 95.67, 122.68, 126.76, 126.96, 129.06, 138.07, 146.12, 149.02, 158.09, 161.49; ESI-MS [M + H]^+^: 287.1. Anal. Calcd for C_14_H_11_ClN_4_O: C, 58.65; H, 3.87; N, 19.54; Found: C, 58.72; H, 3.84; N, 19.57.

*2-((3-Methyl-1-phenyl-1H-pyrazol-5-yl)oxy)benzothiazole* (**6i**), yellow solid, yield 65.7%, m.p. 82–83 °C; IR (KBr, ν_max_, cm^−1^): 2925 (CH_3_), 1558 (-C=N-), 1217 (=C-O-C), 689(-C-S-C). ^1^H-NMR (CDCl_3,_ 400 MHz), δ (ppm): 2.40 (s, 3H, CH_3_), 6.35 (s, 1H, pyrazole-4H), 7.30–7.36 (m, 2H, ArH), 7.43–7.47 (m, 3H, ArH), 7.64–7.66 (d, 2H, *J* = 8.2 Hz, ArH), 7.70–7.72 (d, 1H, *J* = 8.08 Hz, ArH), 7.81-7.83 (d, 1H, *J* = 8.12 Hz, ArH); ^13^C-NMR (CDCl_3,_ 100 MHz), δ (ppm): 14.67, 18.43, 94.90, 121.46, 122.27, 122.92, 124.79, 126.59, 127.24, 129.15, 132.37, 137.79, 147.36, 148.48, 149.04, 168.85; ESI-MS [M + H]^+^: 308.2. Anal. Calcd for C_17_H_13_N_3_OS: C, 66.43; H, 4.26; N, 13.67; Found: C, 66.40; H, 4.23; N, 13.67.

#### 3.2.3. Synthesis of Compounds **7a**–**7e**

Compound **6a** (0.5 mmol) was dissolved in *N*,*N-*Dimethylformamide (DMF, 6 mL) and *N*-bromosuccinimide (NBS, 0.6 mmol) was added in this solution, this mixture was stirred at room temperature overnight. Then, the mixture was poured into water (10 mL) and stored overnight, filtered, and the residue was the desired product **7a**. Compounds **7b**–**7e** were synthesized using the same procedures.

*2-((4-Bromo-1-(2,2,2-trifluoroethyl)-3-methyl-1H-pyrazol-5-yl)oxy)-6-chlorobenzoxazole* (**7a**), white solid, yield 49.8%, m.p. 131–132 °C; IR (KBr, ν_max_, cm^−1^): 2982 (CH_3_), 1625 (-C=N-), 1375 (C-F), 1253 (=C-O-C), 1170 (-C-O-C), 598 (C-Br). ^1^H-NMR (CDCl_3,_ 400 MHz), δ (ppm): 2.64 (s, 3H, CH_3_), 4.93–4.99 (q, 2H, *J* = 8.24 Hz, CH_2_CF_3_), 7.40–7.43 (dd, 1H, *J* = 8.48, 1.8 Hz, ArH), 7.59 (d, 1H, *J* = 1.4 Hz, ArH), 7.61–7.63 (d, 1H, *J* = 8.52 Hz, ArH); ^13^C-NMR (CDCl_3_, 100 MHz), δ (ppm): 14.24, 45.62 (q, ^2^*J*_C,F_ = 35 Hz), 96.28, 111.30, 120.48, 122.85 (q, ^1^*J*_C,F_ = 280 Hz), 126.37, 131.15, 138.46, 149.10, 151.88, 152.43, 163.48; ESI-MS [M + H]^+^: 410, 412. Anal. Calcd for C_13_H_8_BrClF_3_N_3_O_2_: C, 38.03; H, 1.96; N, 10.23; Found: C, 37.96; H, 1.95; N, 10.22.

*2-((4-Bromo-1-(2,2,2-trifluoroethyl)-3-methyl-1H-pyrazol-5-yl)oxy)benzoxazole* (**7b**), white solid, yield 52.2%, m.p. 107–108 °C; IR (KBr, ν_max_, cm^−1^): 2925 (CH_3_), 1622 (-C=N-), 1319 (C-F), 1267 (=C-O-C), 1162 (-C-O-C), 745 (C-Br). ^1^H-NMR (CDCl_3_, 400 MHz), δ (ppm): 2.28 (s, 3H, CH_3_), 4.62–4.68 (q, 2H, *J* = 8.16 Hz, CH_2_CF_3_), 7.31–7.36 (m, 2H, ArH), 7.48–7.50 (m, 1H, ArH), 7.57–7.59 (m, 1H, ArH); ^13^C-NMR (CDCl_3_, 100 MHz), δ (ppm): 13.14, 49.42 (q, ^2^*J*_C,F_ = 36 Hz), 84.38, 110.47, 119.52, 122.34 (q, ^1^*J*_C,F_ = 278 Hz), 124.56, 125.20, 139.90, 144.29, 149.03, 149.05, 158.77; ESI-MS [M + H]^+^: 376.1, 378.1; Anal. Calcd for C_13_H_9_BrF_3_N_3_O_2_: C, 41.51; H, 2.41; N, 11.17; Found: C, 47.47; H, 2.39; N, 11.16.

*2-((4-Bromo-1-(2,2,2-trifluoroethyl)-3-methyl-1H-pyrazol-5-yl)oxy)-5-chlorobenzoxazole* (**7c**), white solid, yield 83.2%, m.p. 112–113 °C; IR (KBr, ν_max_, cm^−1^): 2955 (CH_3_), 1627 (-C=N-), 1349 (C-F), 1253 (=C-O-C), 1172 (-C-O-C), 698 (C-Br). ^1^H-NMR (CDCl_3,_ 400 MHz), δ (ppm): 2.61 (s, 3H, CH_3_), 4.91–4.97 (q, 2H, *J* = 8.36 Hz, CH_2_CF_3_), 7.38–7.40 (dd, 1H, *J* = 8.52, 1.92 Hz, ArH), 7.56–7.57 (d, 1H, *J* = 1.84 Hz, ArH), 7.58–7.61 (d, 1H, *J* = 8.52 Hz, ArH); ^13^C-NMR (CDCl_3_, 100 MHz), δ (ppm): 13.13, 49.61 (q, ^2^*J*_C,F_ = 36 Hz), 84.38, 110.47, 118.18, 122.39 (q, ^1^*J*_C,F_ = 278 Hz), 124.20, 130.25, 134.33, 145.97, 146.82, 148.91, 168.23; ESI-MS [M + H]^+^: 410.1, 412.1. Anal. Calcd for C_13_H_8_BrClF_3_N_3_O_2_: C, 38.03; H, 1.96; N, 10.23. Found: C, 37.96; H, 1.95; N, 10.22.

*2-((4-Bromo-1-(2,2,2-trifluoroethyl)-3-methyl-1H-pyrazol-5-yl)oxy)-5-chloropyrimidine* (**7d**), white solid, yield 86.4%, m.p. 101–102 °C; IR (KBr, ν_max_, cm^−1^): 2993(CH_3_), 1562 (-C=N-), 1279 (C-F), 1160 (=C-O-C), 641 (C-Br). ^1^H-NMR (CDCl_3_, 400 MHz), δ (ppm): 2.27 (s, 3H, CH_3_), 4.54–4.60 (q, 2H, *J* = 8.24 Hz, CH_2_CF_3_), 8.55 (s, 2H, pyrimidine-4,6H); ^13^C-NMR (CDCl_3,_ 100 MHz), δ (ppm): 13.12, 49.27 (q, ^2^*J*_C,F_ = 36 Hz), 84.73, 122.42 (q, ^1^*J*_C,F_ = 278 Hz), 127.61, 145.12, 148.72, 158.34, 160.60; ESI-MS [M + H]^+^: 371, 373. Anal. Calcd for C_10_H_7_BrClF_3_N_4_O: C, 32.33; H, 1.90; N, 15.08. Found: C, 32.26; H, 1.88; N, 15.05.

*5-(Benzyloxy)-4-bromo-3-methyl-1-phenyl-1H-pyrazole* (**7e**), yellow liquid, yield 35.4%; ^1^H-NMR (CDCl_3_, 400 MHz), δ (ppm): 2.30 (s, 3H, CH_3_), 5.18 (s, 2H, CH_2_), 7.25–7.33 (m, 6H, ArH), 7.37–7.42 (td, 2H, *J* = 7.4, 1.88 Hz, ArH), 7.53–7.54 (d, 1H, *J* = 0.88 Hz, ArH), 7.55–7.56 (m, 1H, ArH); ^13^C-NMR (CDCl_3_, 100 MHz), δ (ppm): 13.15, 75.96, 82.42, 122.62, 126.89, 128.49, 128.64, 128.76, 128.91, 134.99, 138.36, 147.49, 149.84; ESI-MS [M + H]^+^: 343, 345. Anal. Calcd for C_17_H_15_BrN_2_O: C, 59.49; H, 4.41; N, 8.16. Found: C, 59.30; H, 4.36; N, 8.14.

### 3.3. Biological Evaluation

#### 3.3.1. Inhibitory Effect of the Target Compounds on the Growth of Weed Roots and Shoots

Solutions of 1 g/L and 10 g/L of the tested compounds in DMF were prepared. Agar powder (5 g) was put into boiling distilled water (1 L) until it dissolved, and then cooled down to 40–50 °C. The solution (0.2 mL) containing testing compound and melting agar (19.8 mL) was mixed, and this mixture was added to a 120 mL cup with 4.5 cm diameter. The agar plate without test compound was used as an untreated control. The 15 seeds of *B. campestris*, *A. retroflexus, P. oleracea, P. alopecuroides, E. crus-galli*, and *D. sanguinalis* were put on the surface of the agar plate. These cups were covered with glass lids, and the cultivation conditions were kept at 25 ± 1 °C, 50%–55% relative humidity, and 12 h in the light and 12 h in the dark alternating for seven days. The experiments were conducted in three replicates. Seven days later, the roots lengths of *B. campestris*, *A. retroflexus*, *P. oleracea*, and *D. sanguinalis* and the shoots lengths of *P. alopecuroides*, and *E. crus-galli* were measured. The growth inhibitory rate related to untreated control was determined.

#### 3.3.2. Treatment in Greenhouse Conditions

Twenty seeds of test plants were planted (0.6 cm depth) in plastic boxes (9 cm diameter) containing sandy soil. The plastic boxes were placed at 22–25 °C in a greenhouse. The experiments were conducted in three replicates. The seedlings (one leaf and one stem) of the dicotyledonous weed and the seedlings (two leaf and one stem) of the monocotyledonous weed were sprayed with the test compounds at the concentration of 750 g a. i. ha^−1^. The emulsions of tested compounds were prepared by dissolving them in DMF with the addition proper water contained 0.1% Triton X-100. The fresh weights were determined 15 days later, and the percentage inhibition relative to the water-sprayed controls was calculated.

#### 3.3.3. Inhibitory Effect of the Compound **6d** on Chlorophyll of Weeds

To evaluate the bleaching activity of compound **6d**, the changes in chlorophyll contents of treated seedlings were evaluated by Arnon’s method [34] as modified by Lichtenthaler [35]. Bleached seedlings of *B. campestris, P. alopecuroides, E. crus-galli*, and *D. sanguinalis* with different concentrations were obtained by the method described above. Chlorophyll a and b were extracted from 50 mg of leaf tissue per treatment in 8 mL of 80% acetone in water for 24 h. The absorbance was measured at 663 nm and 646 nm, respectively. The contents of chlorophyll a and b in leaf tissue were finally calculated by the following formula: for chlorophyll a, *C*a = 12.21*A*_663_ − 2.81*A*_646_; for chlorophyll b, *C*_b_ = 20.13*A*_646_ − 5.03*A*_663_. The concentration causing 50% inhibition (IC_50_) of *in vitro* activity for the selected compounds were obtained by analyzing inhibition curves of the activity values (%) versus the logarithm of inhibitory concentration. Diflufenican was selected as the positive control. At least five doses in the inhibitory range were considered, and three replicates were performed under the same conditions. 

## 4. Conclusions

In conclusion, the design, synthesis, and structure–activity relationships of a series of pyrazoyl derivatives have been described. Some compounds displayed an efficient bleaching effect and herbicidal activities against monocotyledonous and dicotyledonous weeds. The herbicidal tests showed that when the 5-position of the pyrazole ring was substituted by heterocycle, the corresponding compounds presented herbicidal activities, especially compound **6d** possessed good herbicidal activity against *D. sanguinalis* at the dosage of 750 g a. i. ha^−1^.

## Supplementary Materials

The following ^1^H-NMR and ^13^C-NMR spectra are available online at http://www.mdpi.com/1420-3049/21/1/39/s1.
