# Supplementary material for: Synthesis and Herbicidal Activity of 5-Heterocycloxy-3-methyl-1-substituted-1H-pyrazoles
Source: Molecules. 2015 Dec 25;21(1):39. doi: 10.3390/molecules21010039 (PMC6273275; doi:10.3390/molecules21010039)
Supplement: Supplementary file 1 [file molecules-21-00039-s001.pdf]

Supplementary Materials: Synthesis and Herbicidal Activity of 5-Heterocycloxy-3-methyl-1-substituted-1*H*-pyrazoles

Jing Kang [1], Xia Li Yue [1], Chang Shui Chen [1], Jian Hong Li [2] and Hong Ju Ma [2,*]

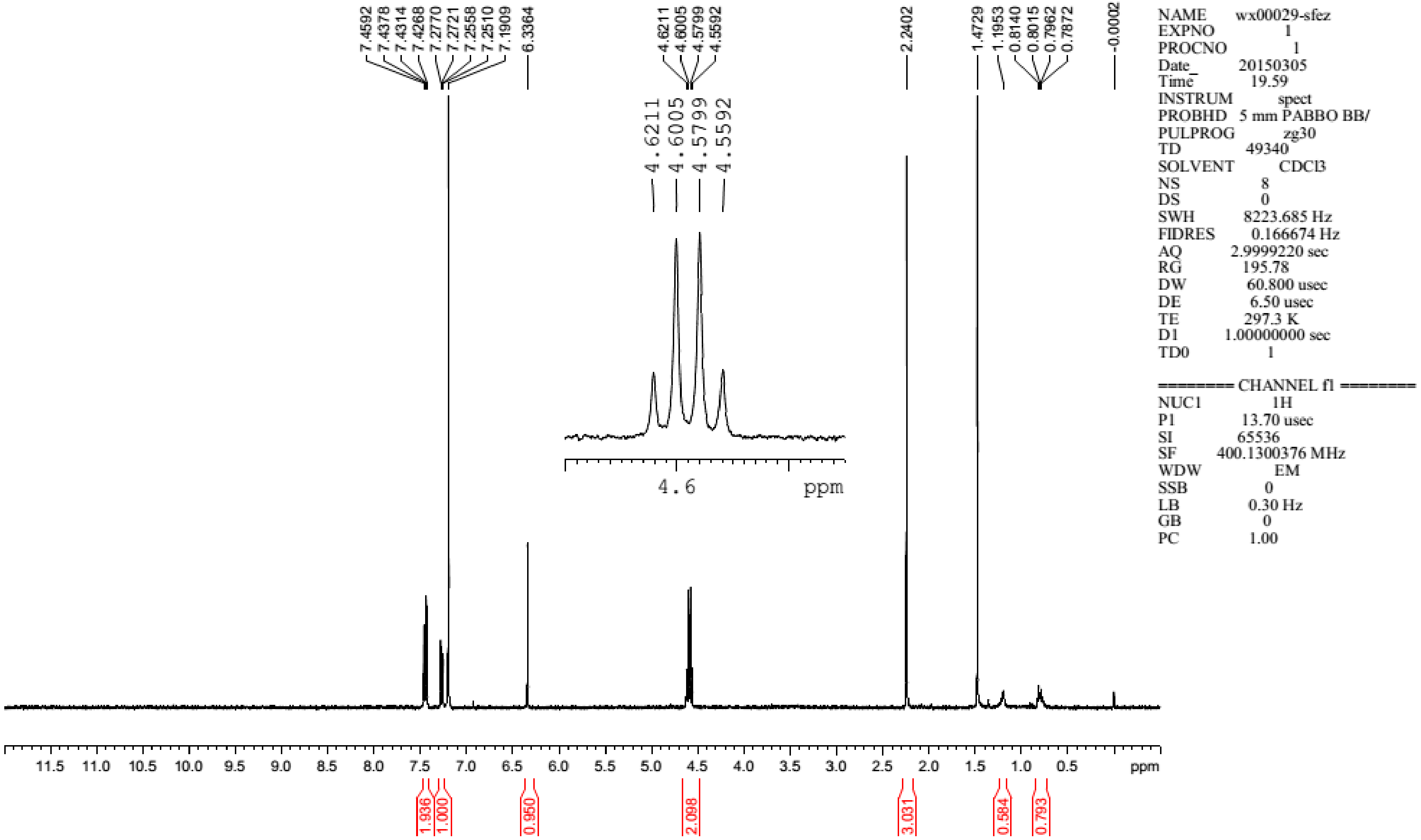

Figure S1. ^{1}H-NMR spectrum of **6a**.

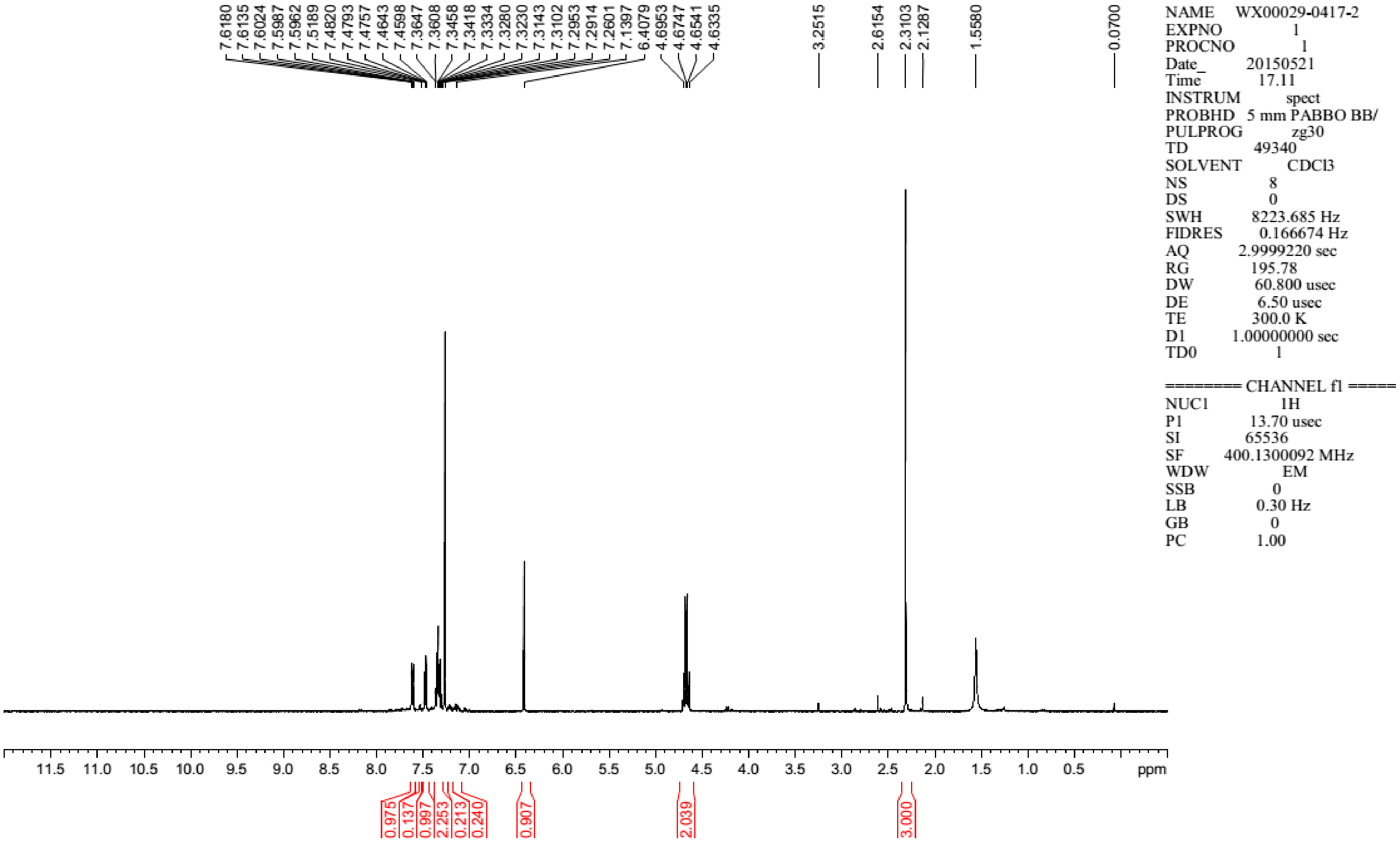

Figure S2. ^{1}H-NMR spectrum of **6b**.

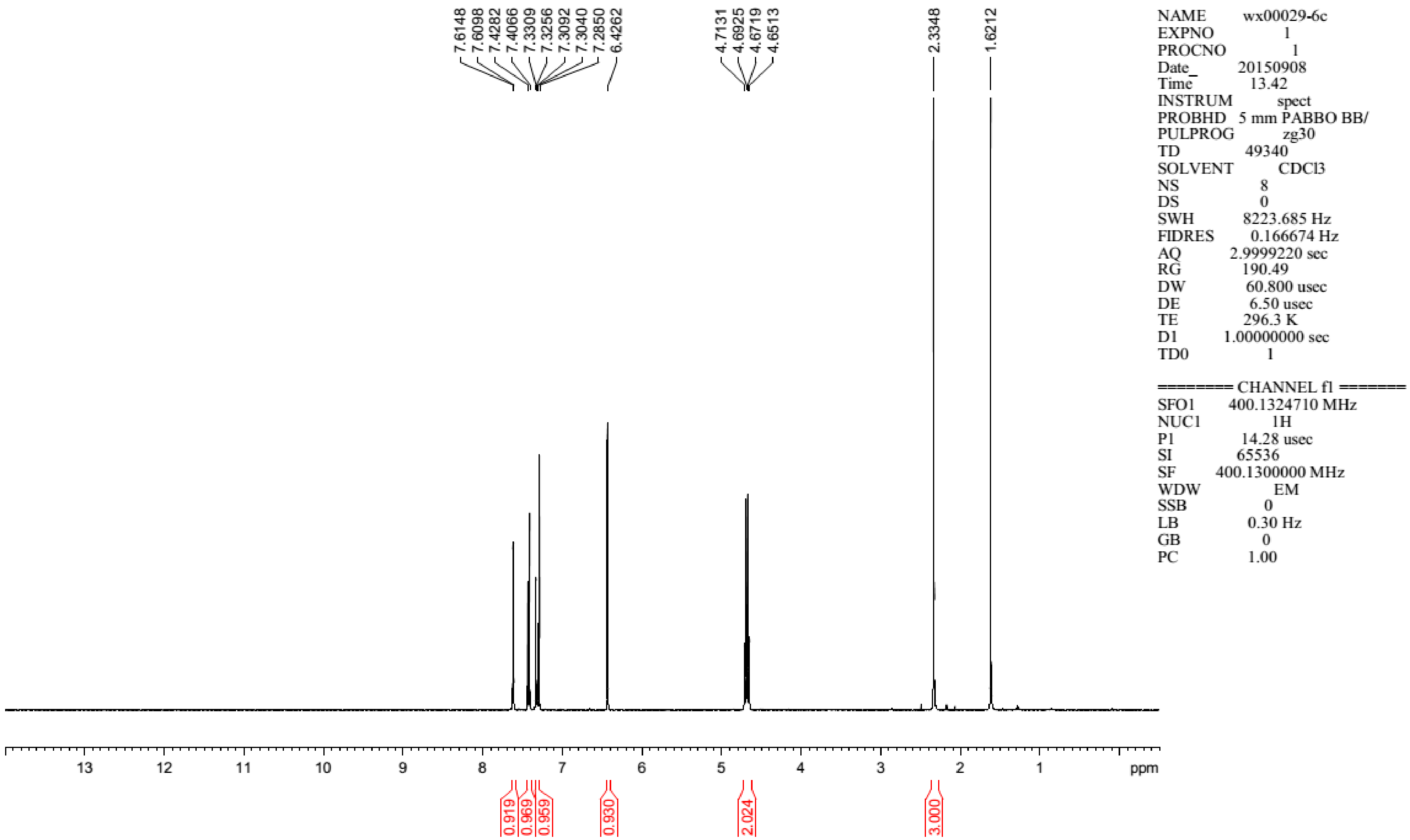

Figure S3. ^{1}H-NMR spectrum of **6c**.

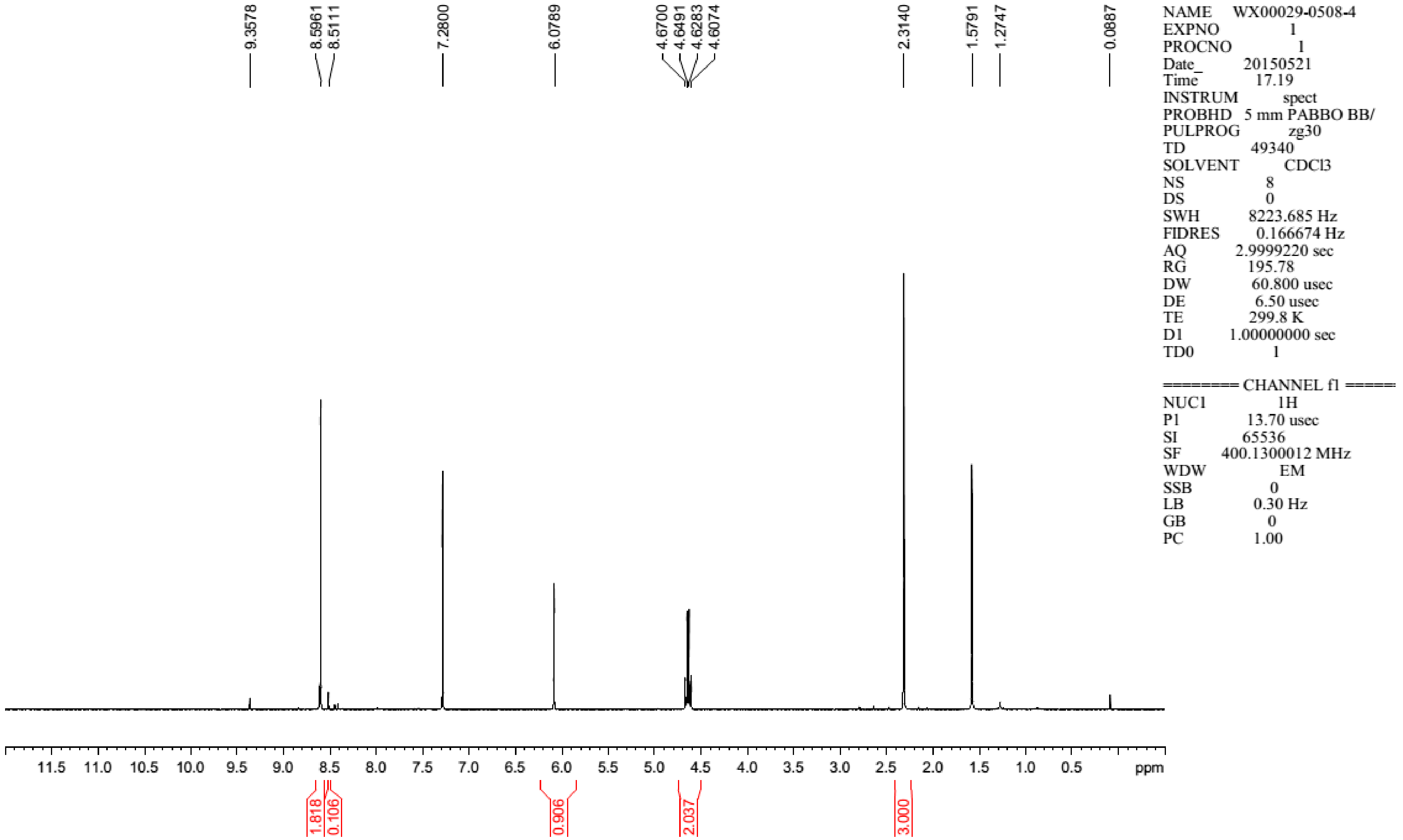

Figure S4. ^{1}H-NMR spectrum of **6d**.

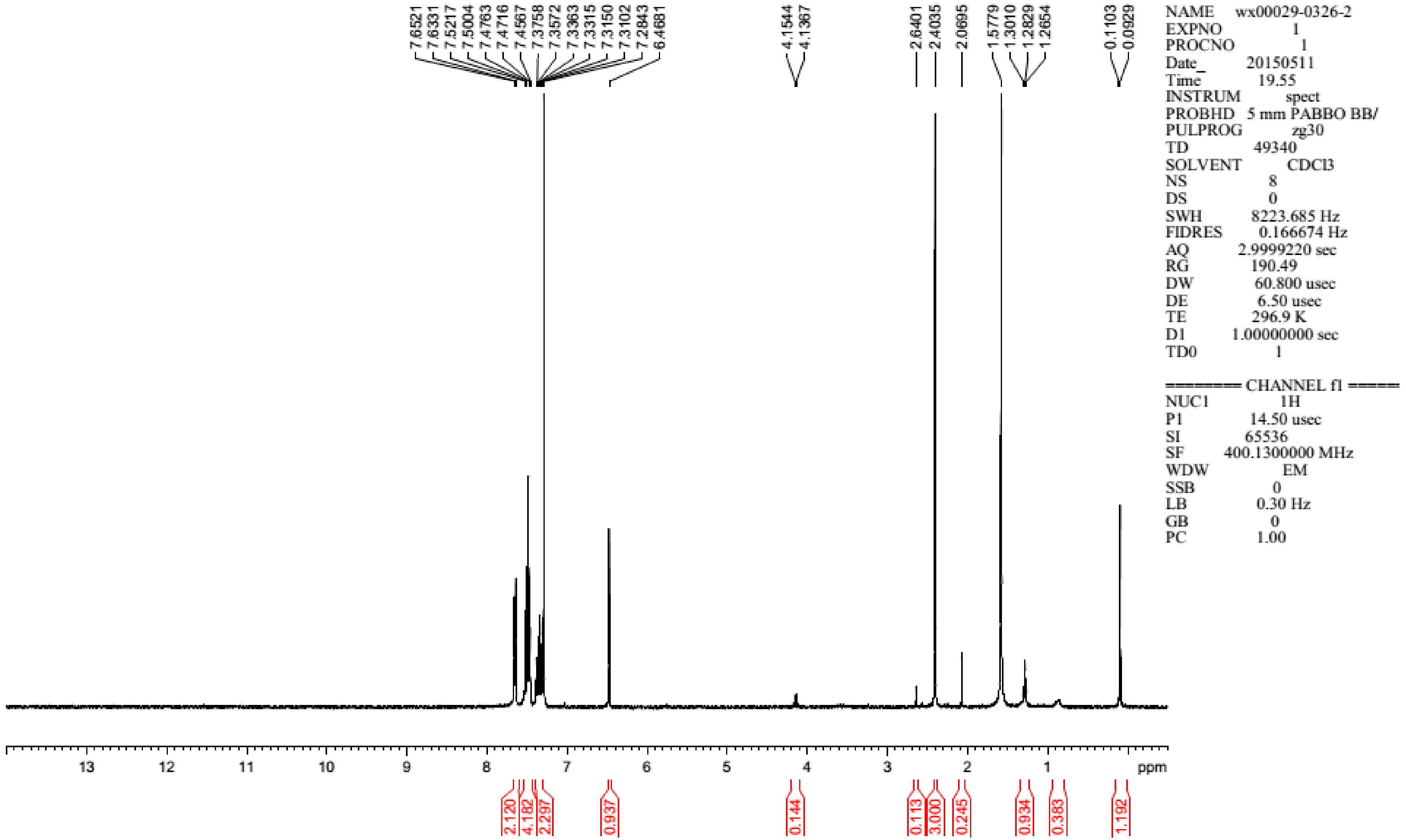

Figure S5. ^{1}H-NMR spectrum of **6e**.

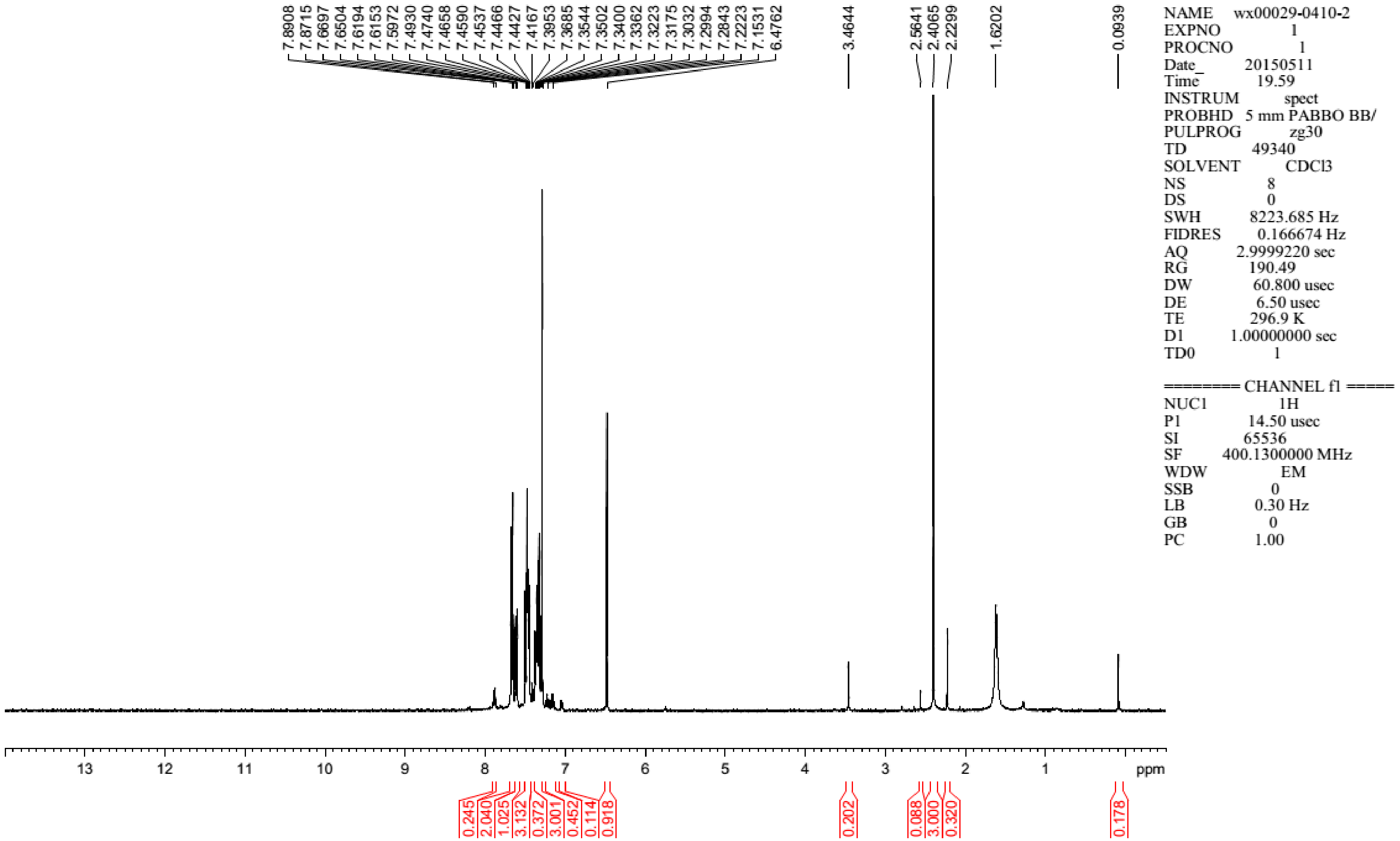

Figure S6. ^{1}H-NMR spectrum of **6f**.

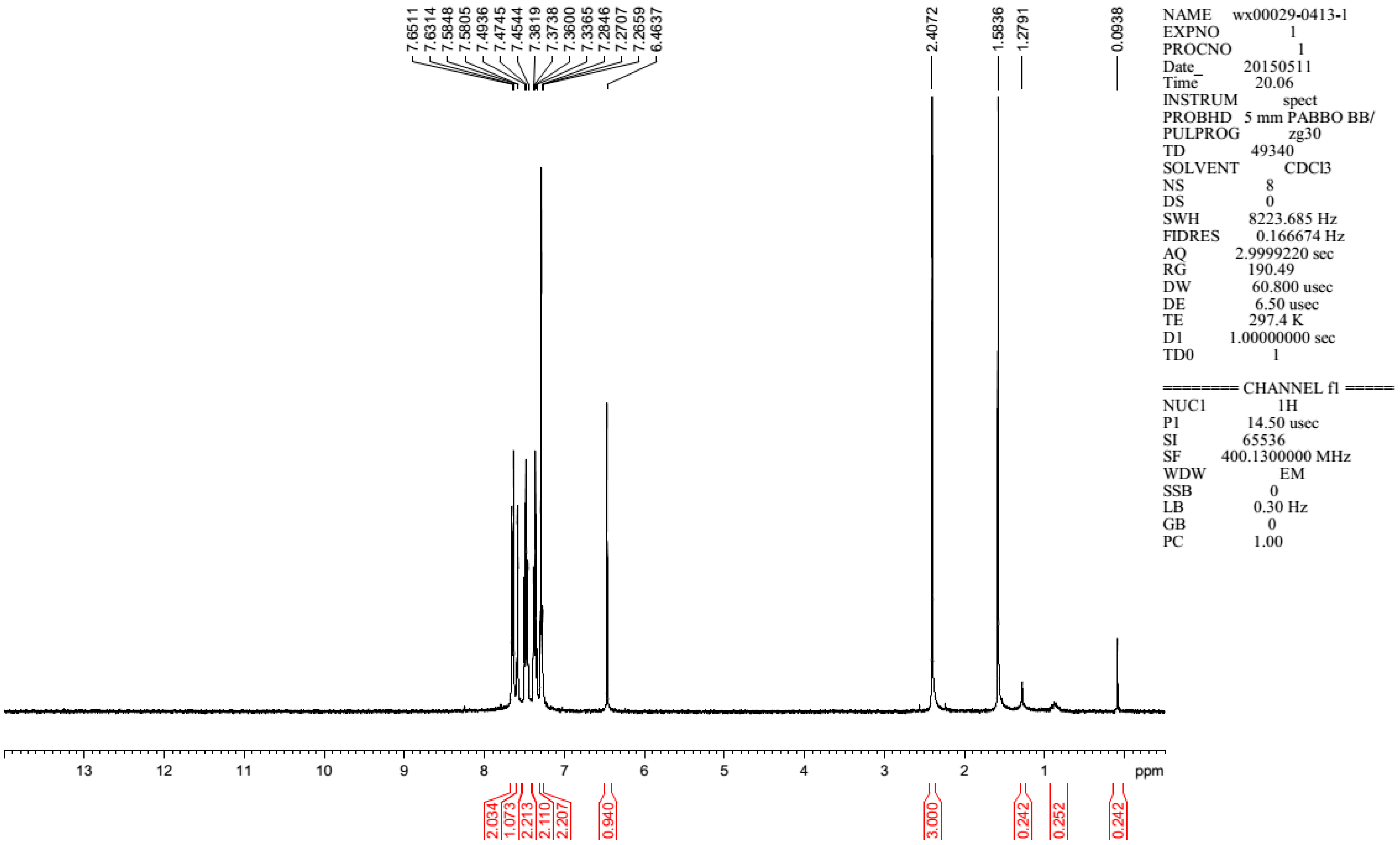

Figure S7. ^{1}H-NMR spectrum of **6g**.

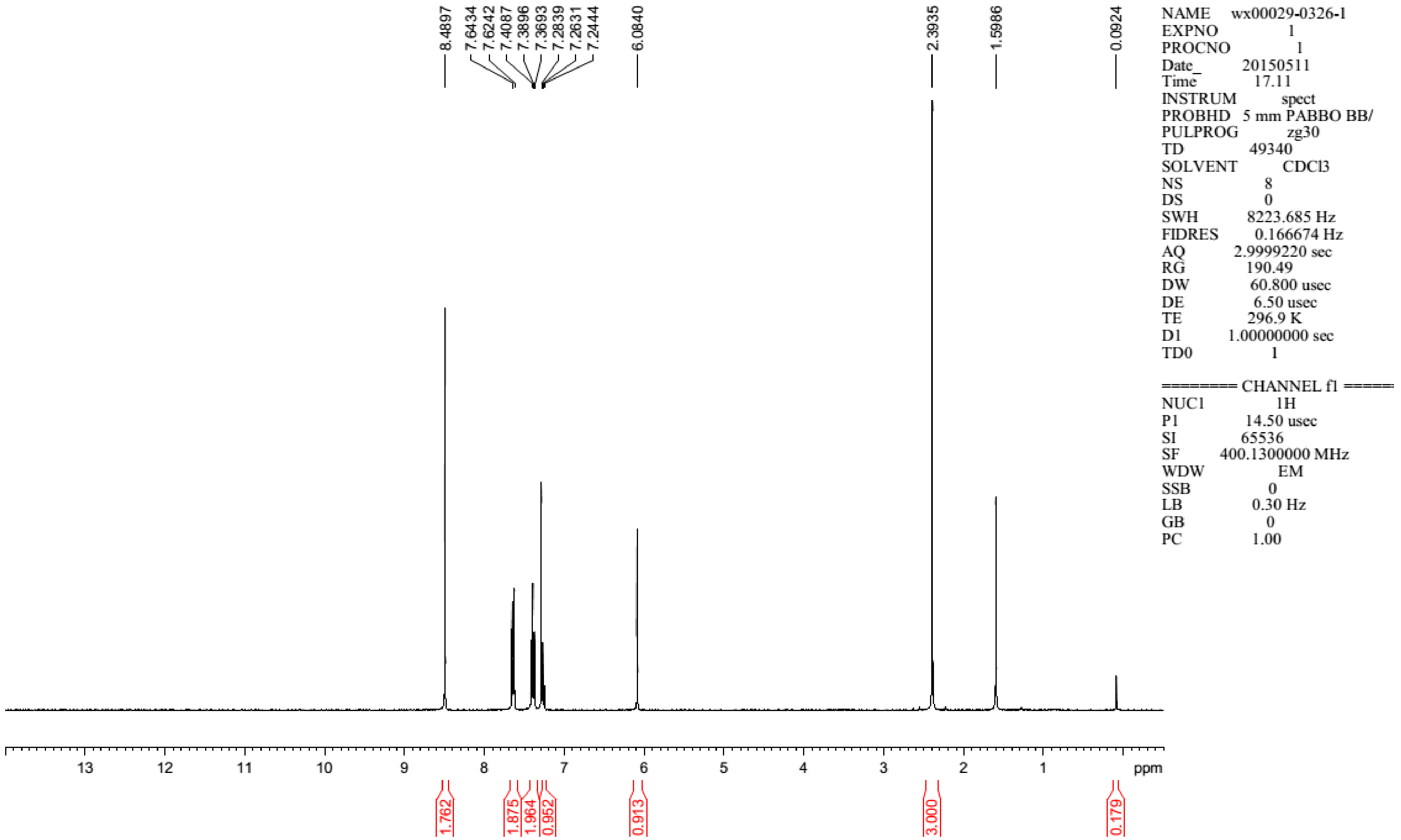

Figure S8. ^{1}H-NMR spectrum of **6h**.

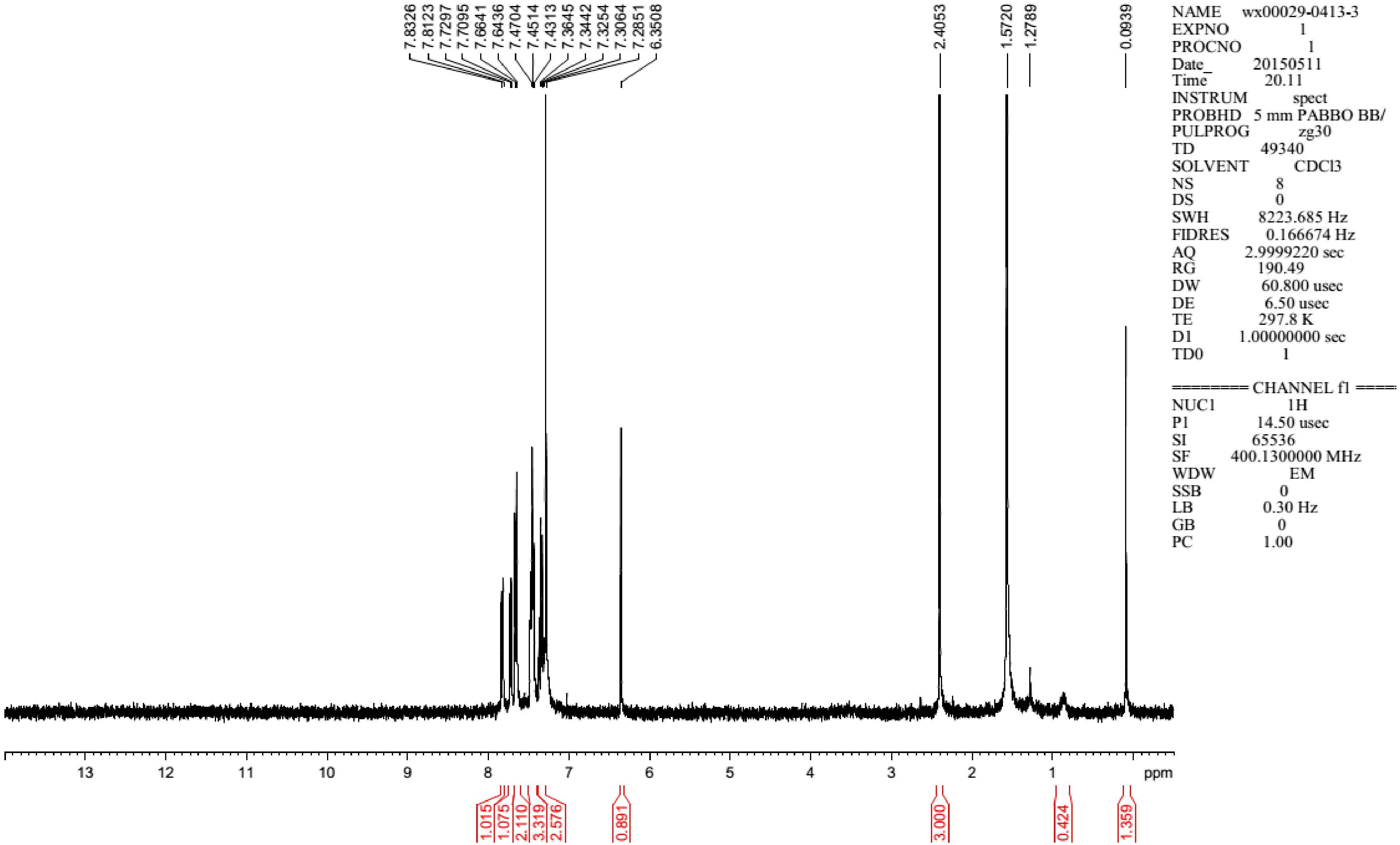

Figure S9. ^{1}H-NMR spectrum of **6i**.

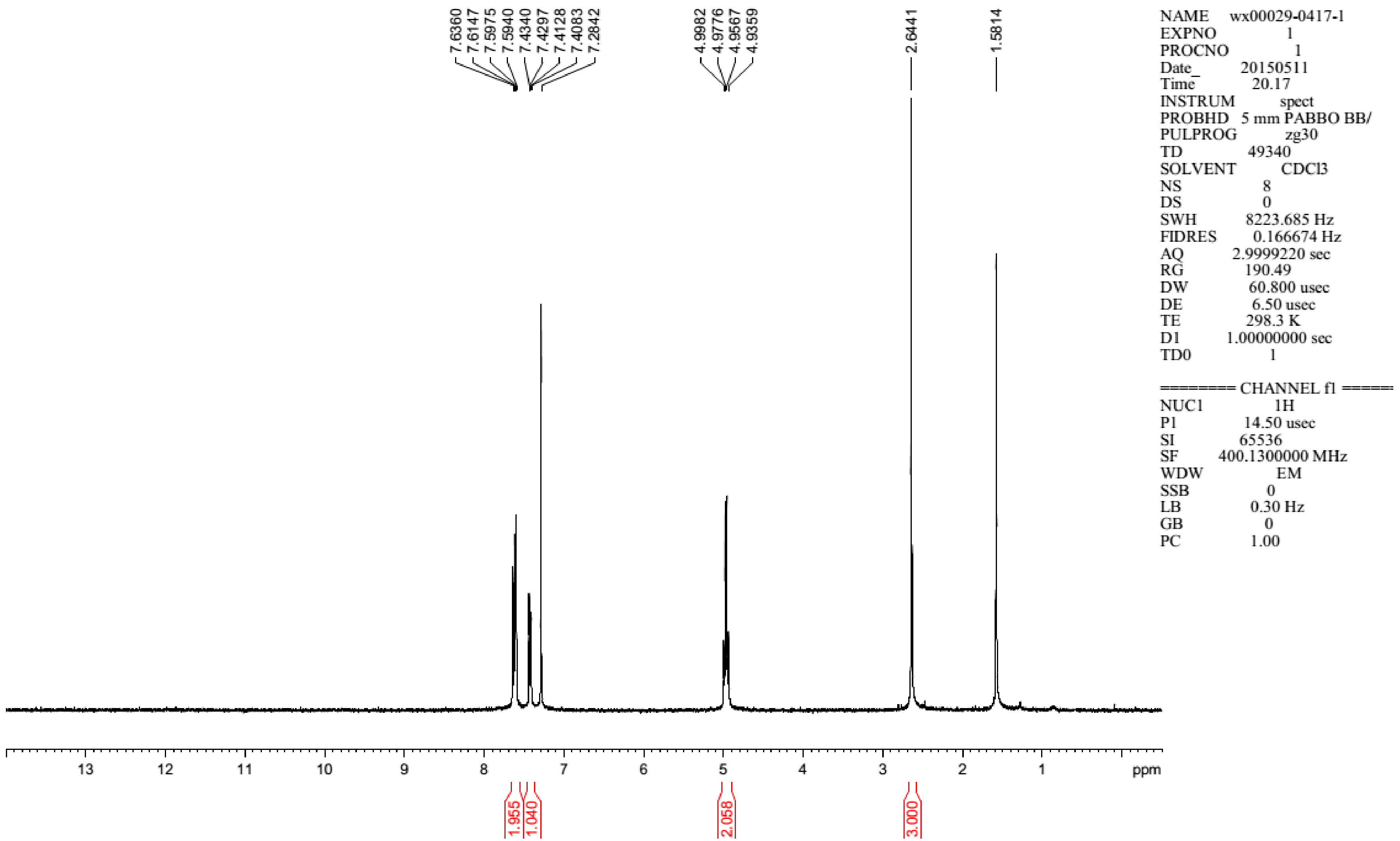

Figure S10. ^{1}H-NMR spectrum of **7a**.

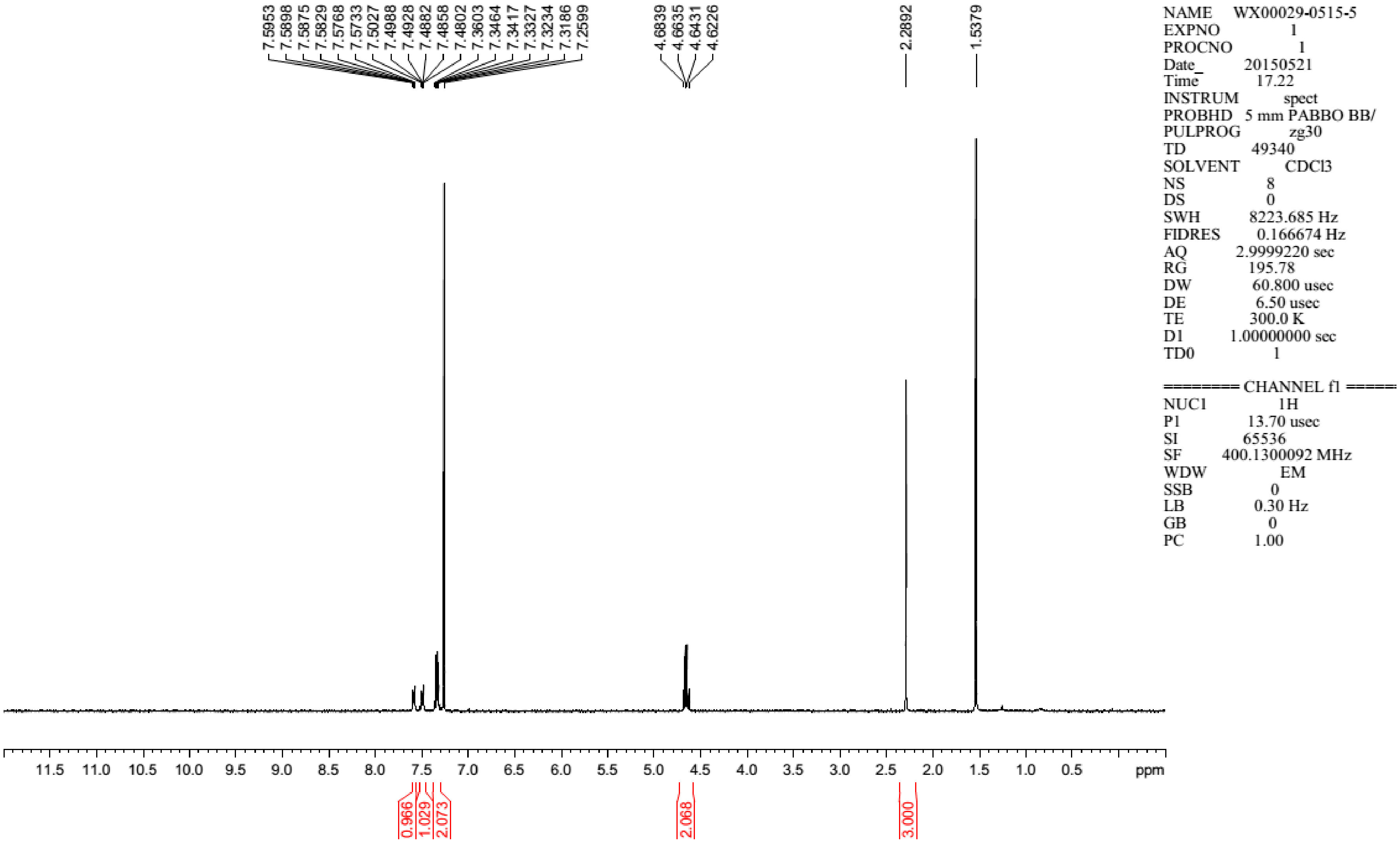

Figure S11. ^{1}H-NMR spectrum of **7b**.

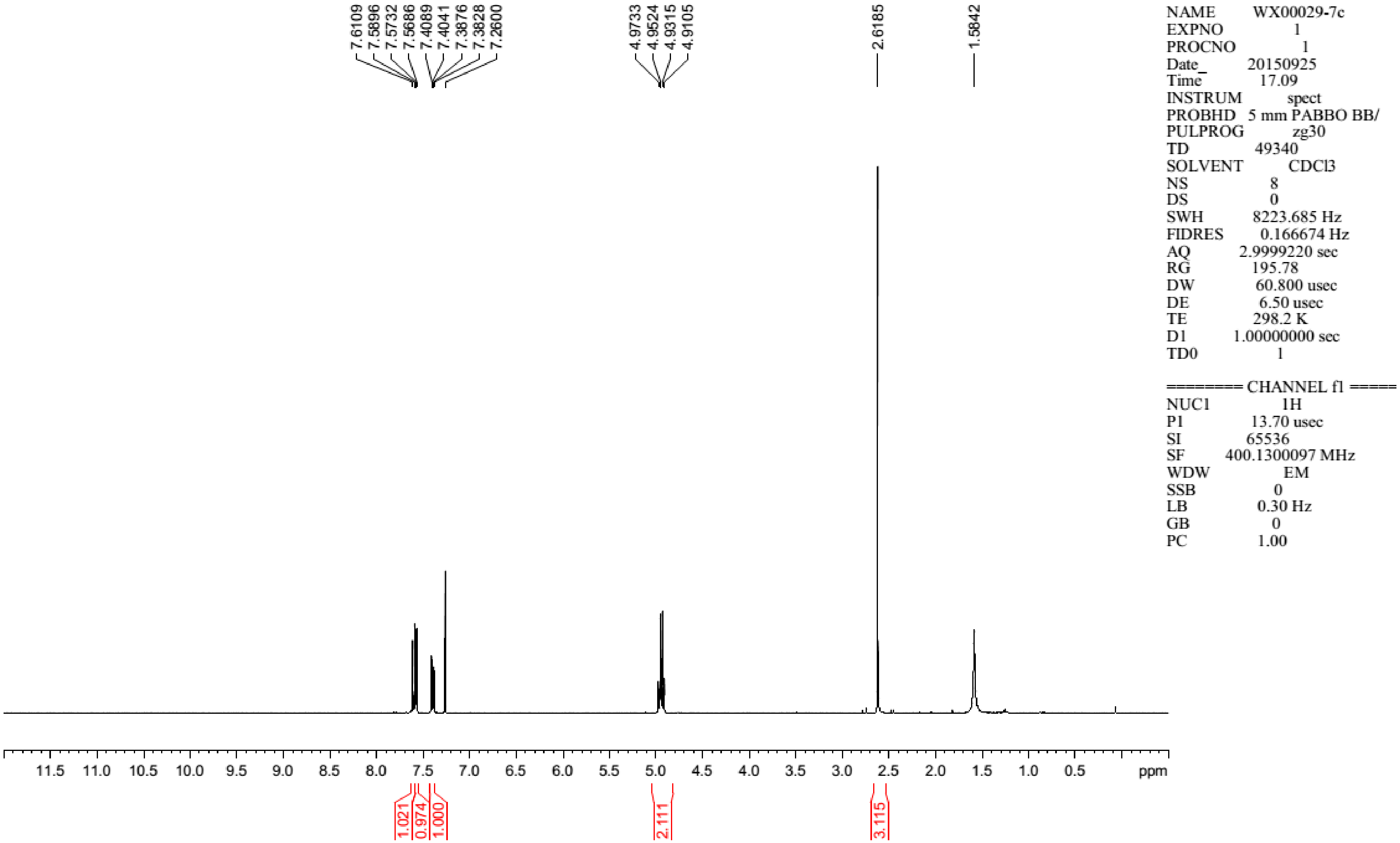

Figure S12. ^{1}H-NMR spectrum of **7c**.

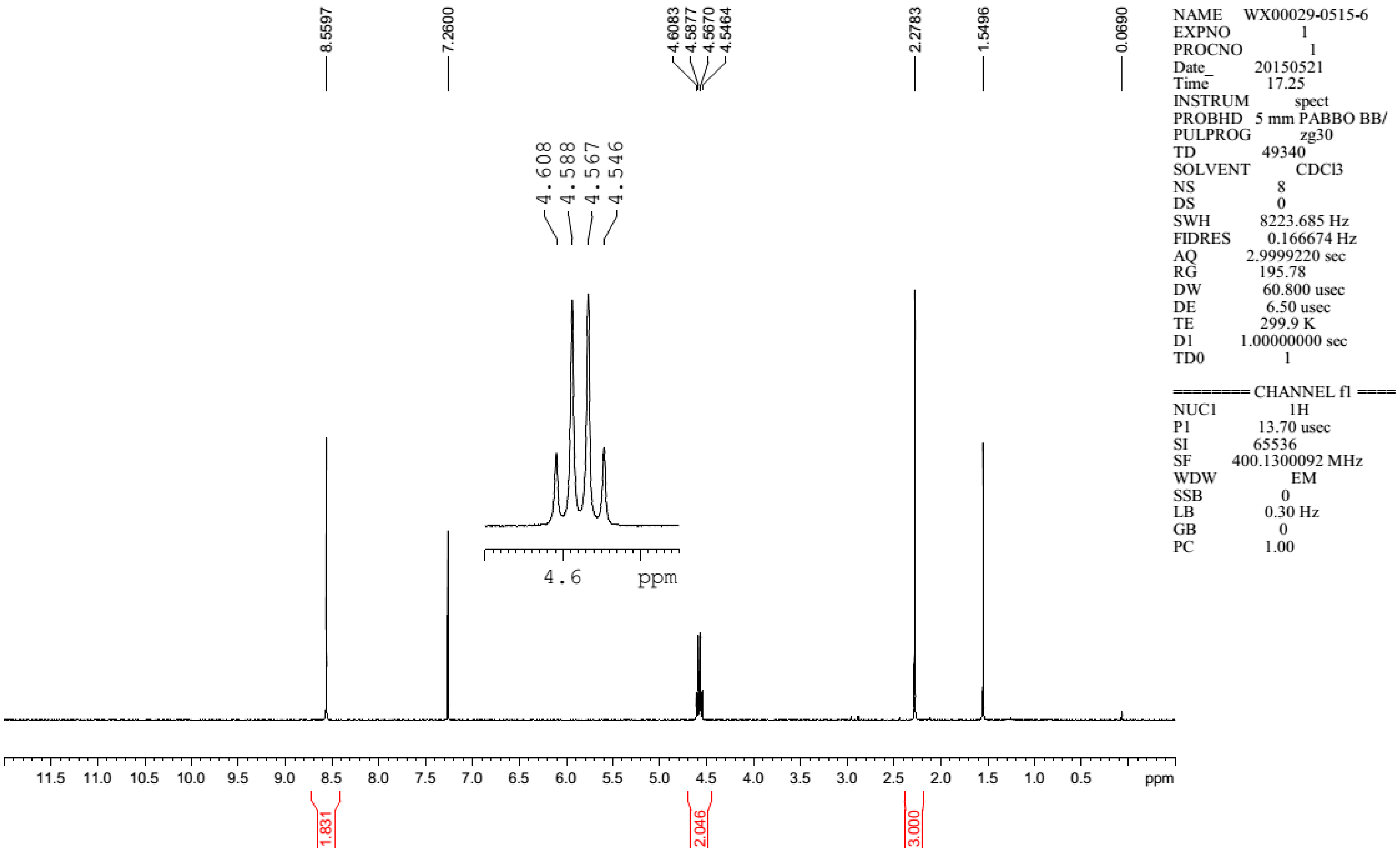

Figure S13. ^{1}H-NMR spectrum of **7d**.

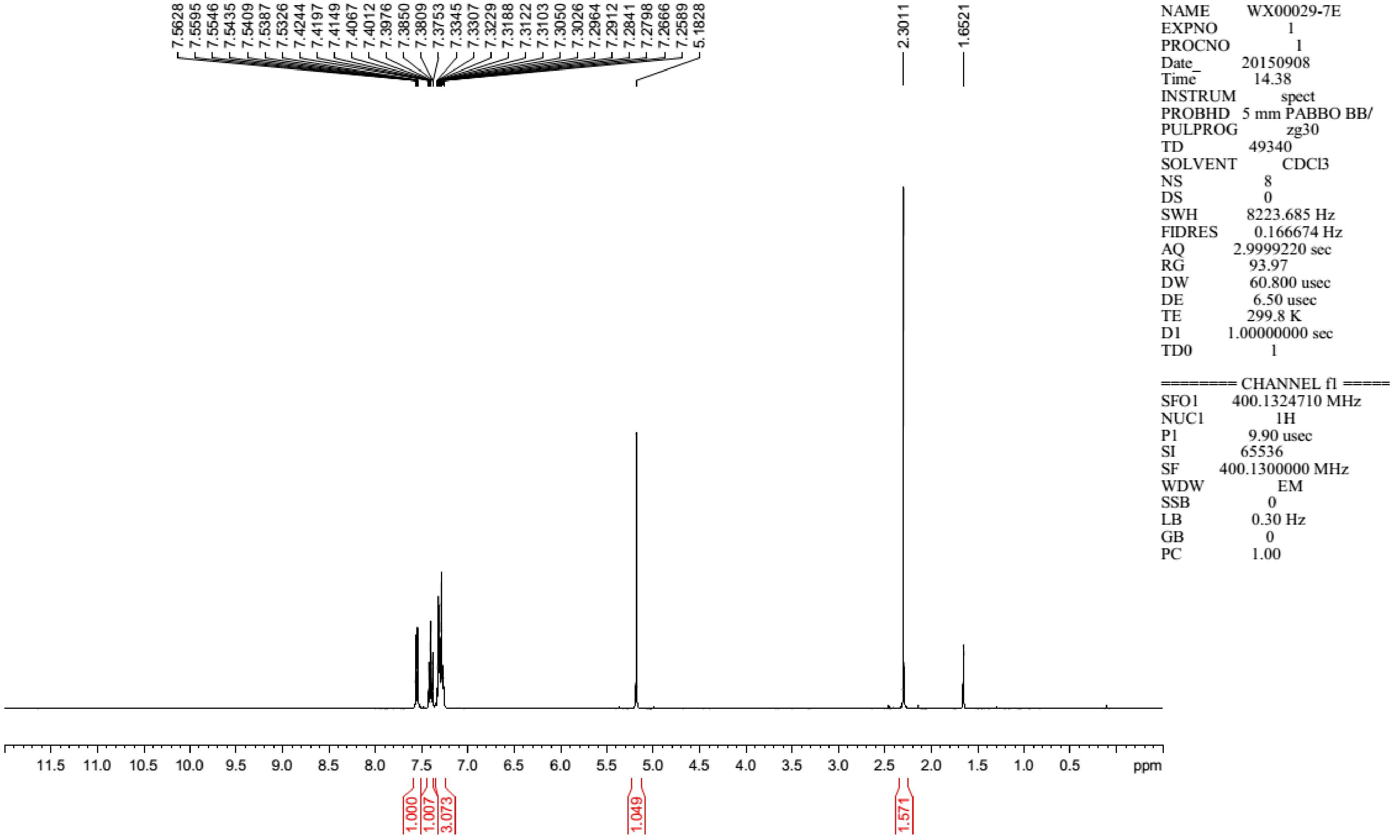

Figure S14. ^{1}H-NMR spectrum of **7e**.

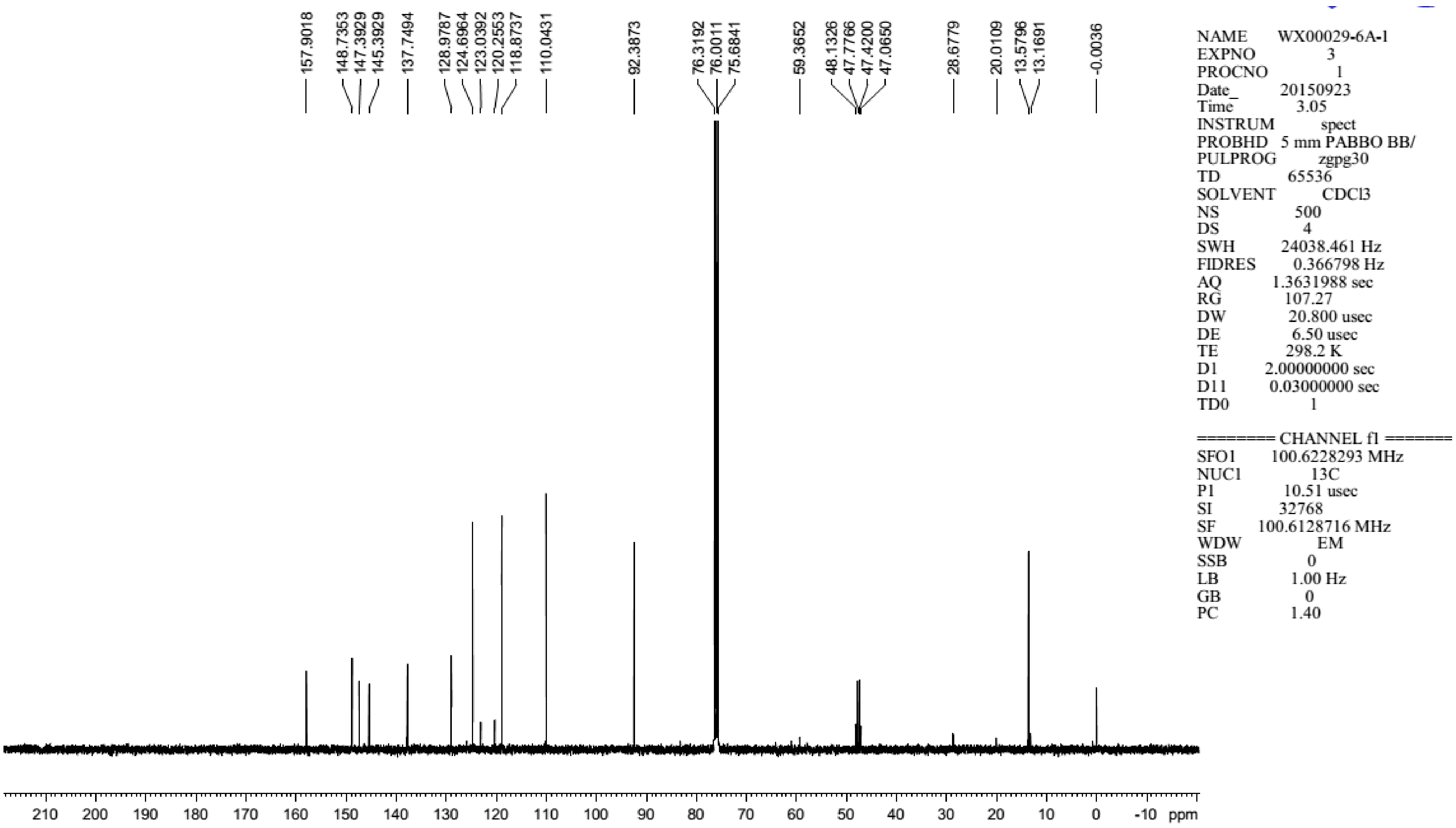

Figure S15. ^{13}C-NMR spectrum of **6a**.

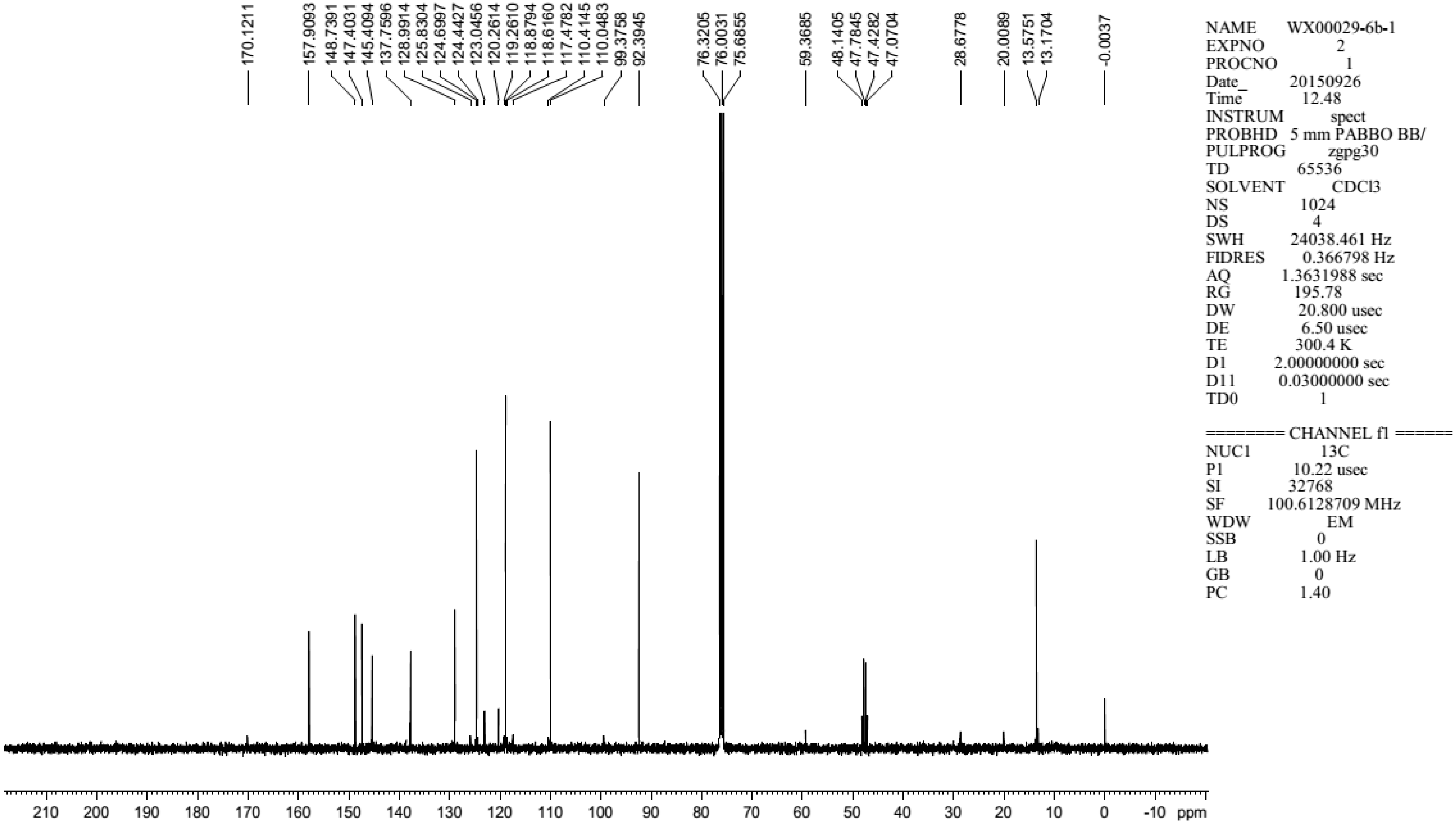

Figure S16. ^{13}C-NMR spectrum of **6b**.

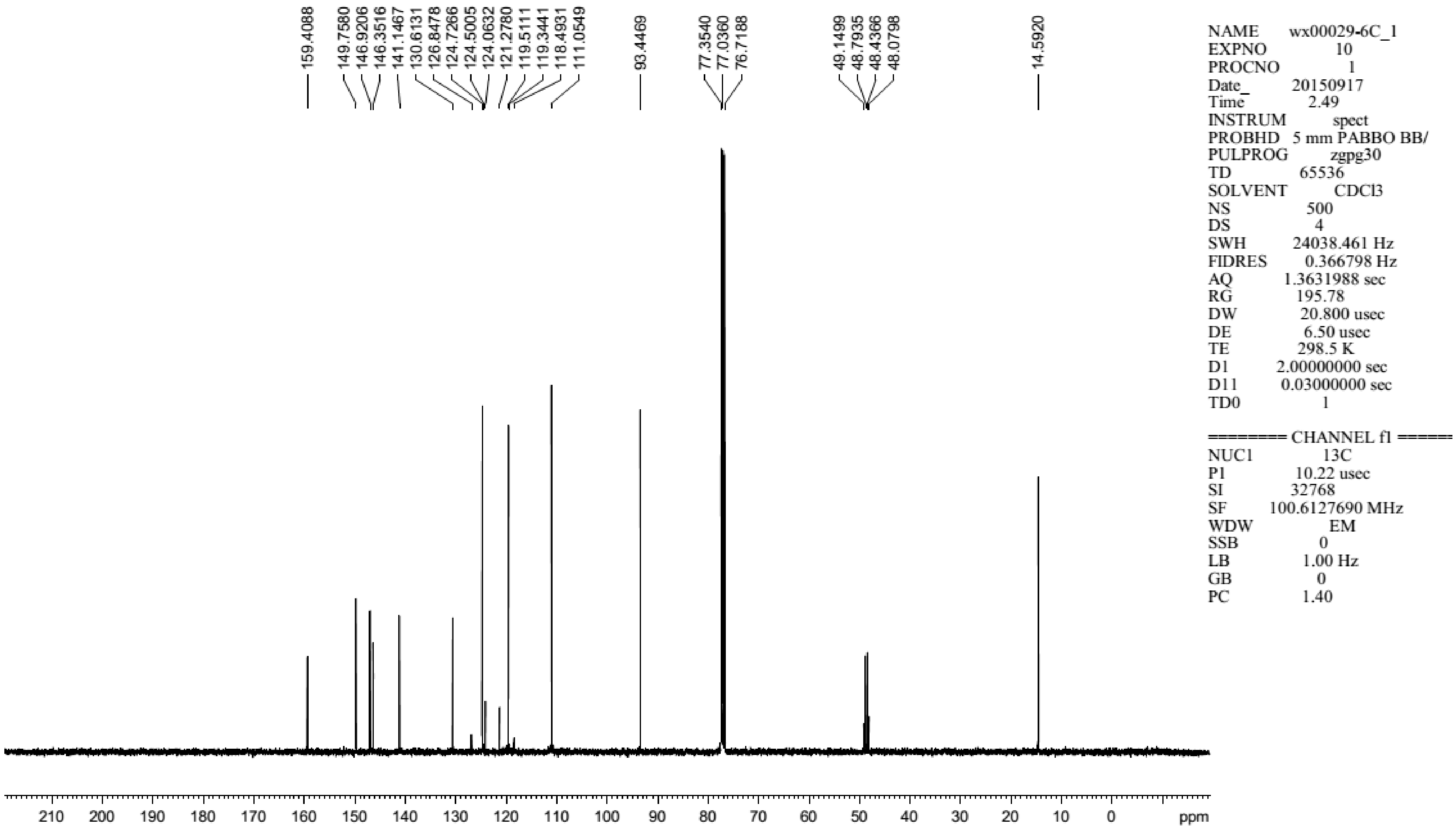

Figure S17. ^{13}C-NMR spectrum of **6c**.

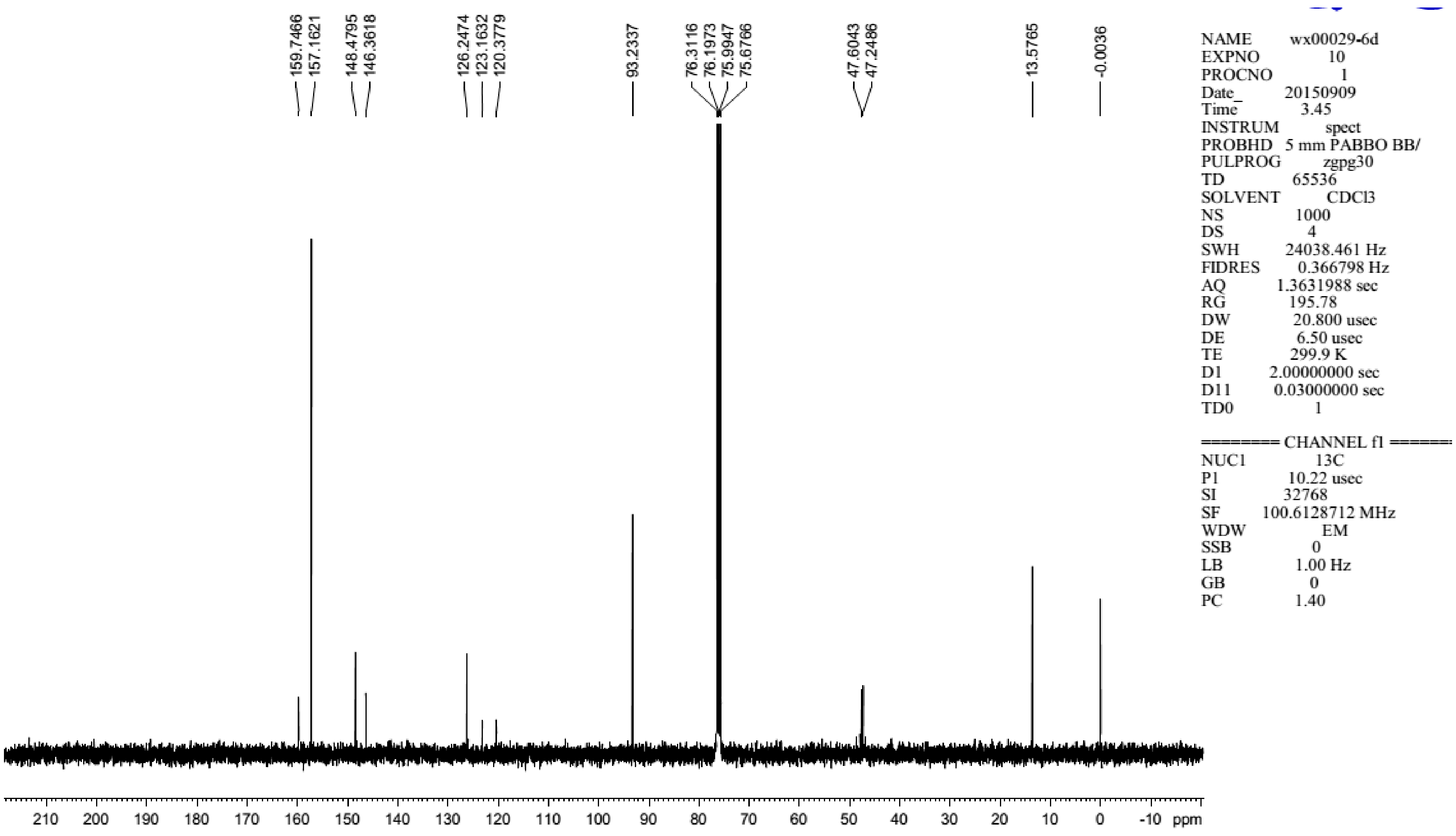

Figure S18. ^{13}C-NMR spectrum of **6d**.

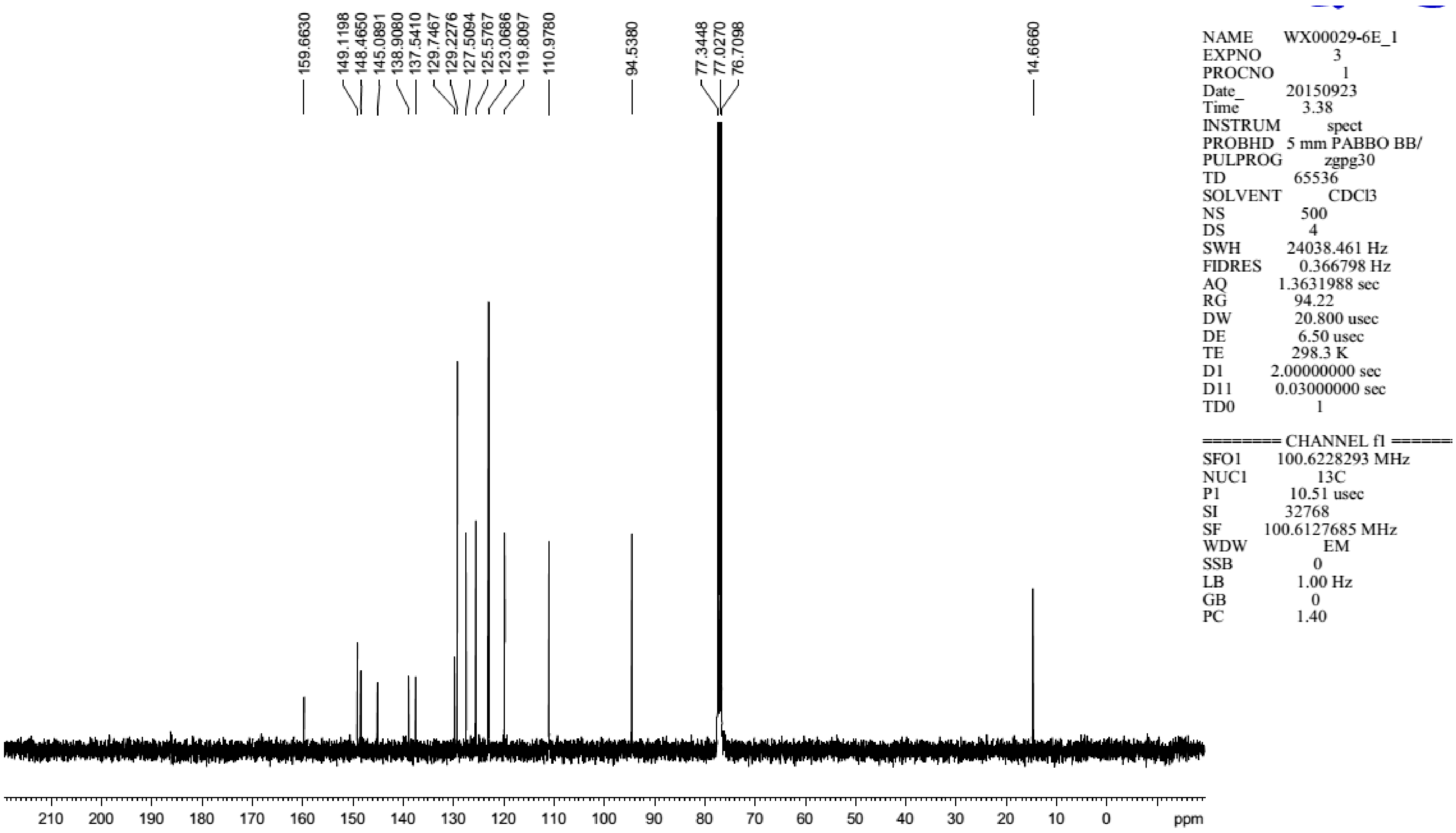

Figure S19. ^{13}C-NMR spectrum of **6e**.

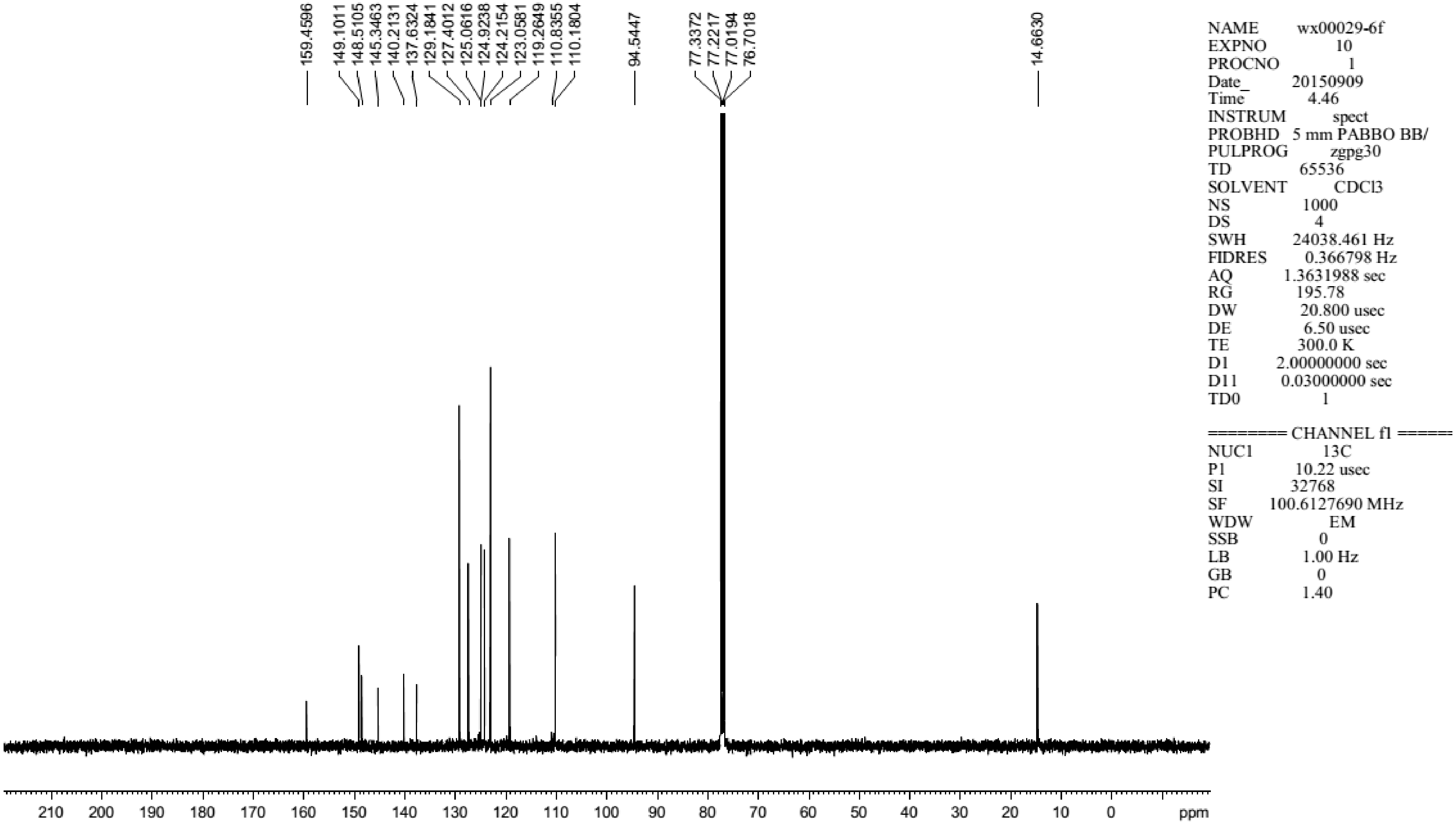

Figure S20. ^{13}C-NMR spectrum of **6f**.

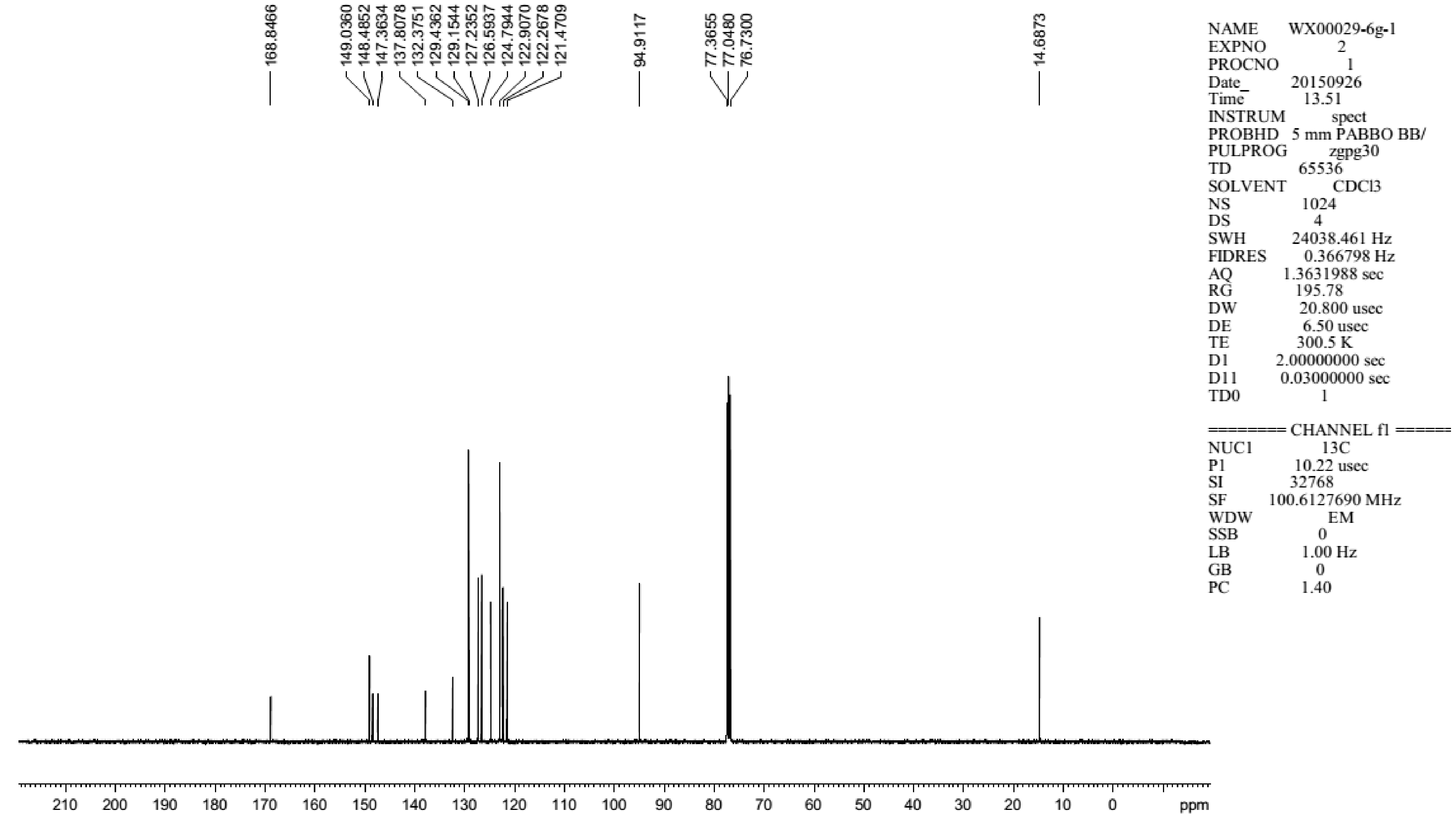

Figure S21. ^{13}C-NMR spectrum of **6g**.

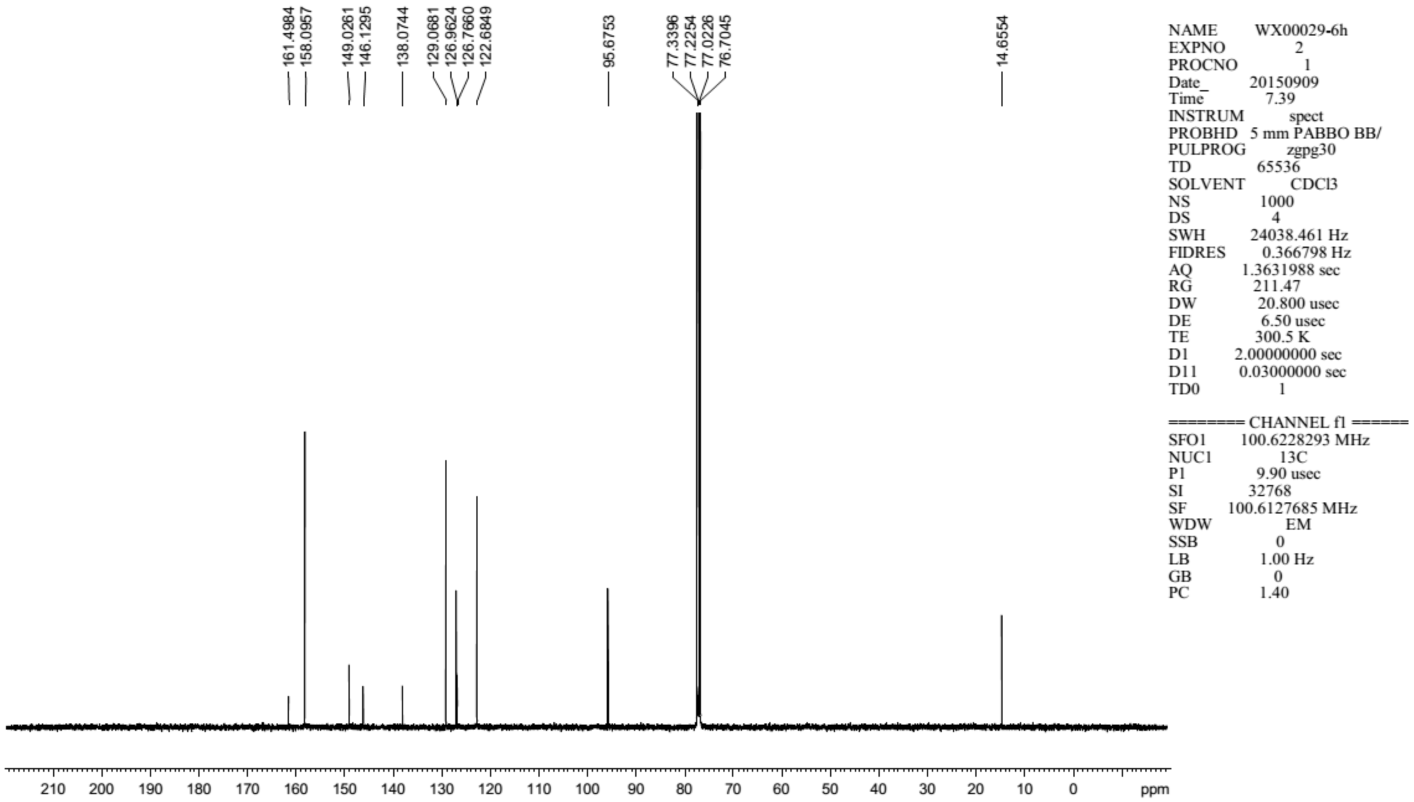

Figure S22. ^{13}C-NMR spectrum of **6h**.

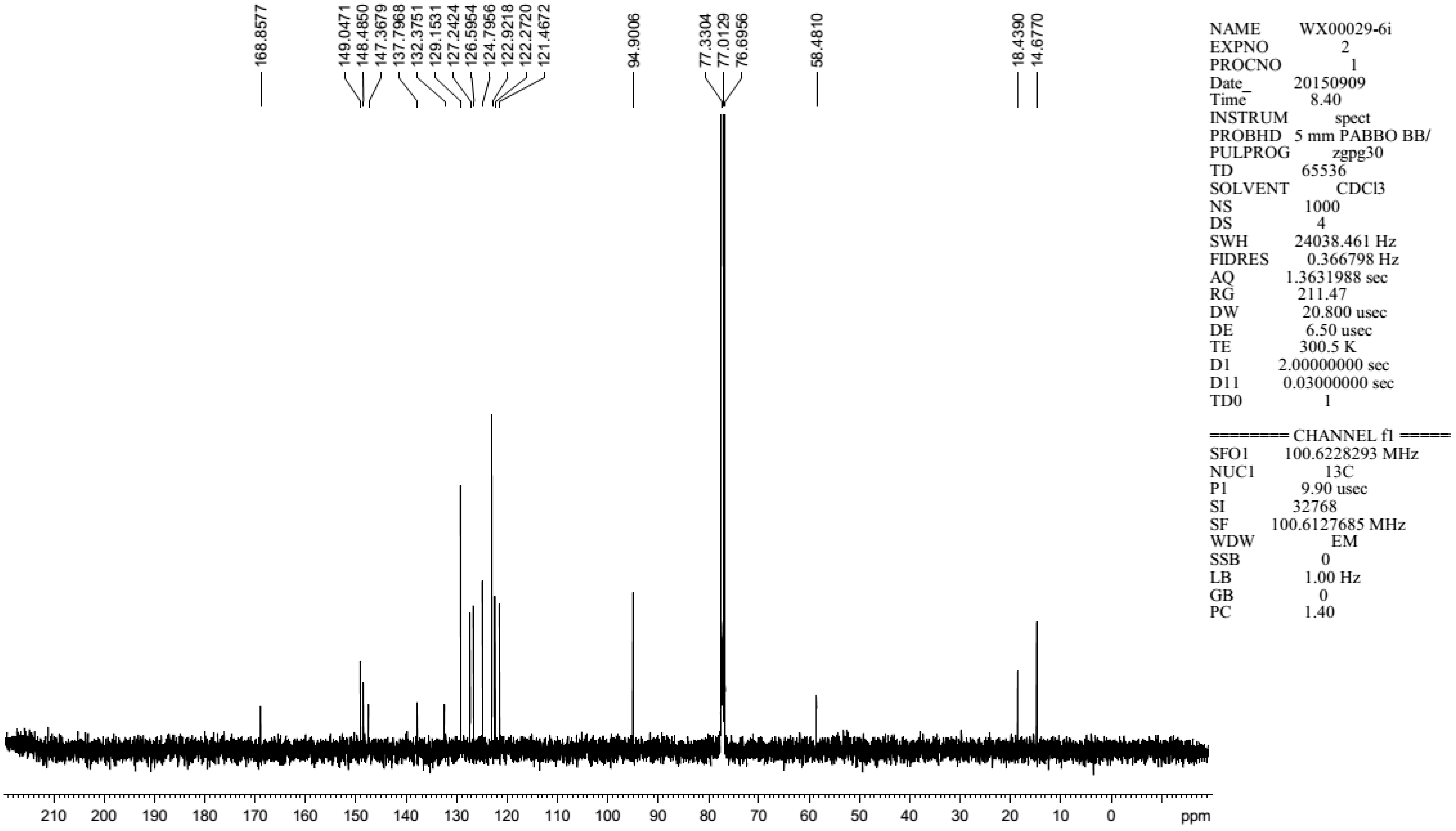

Figure S23. ^{13}C-NMR spectrum of **6i**.

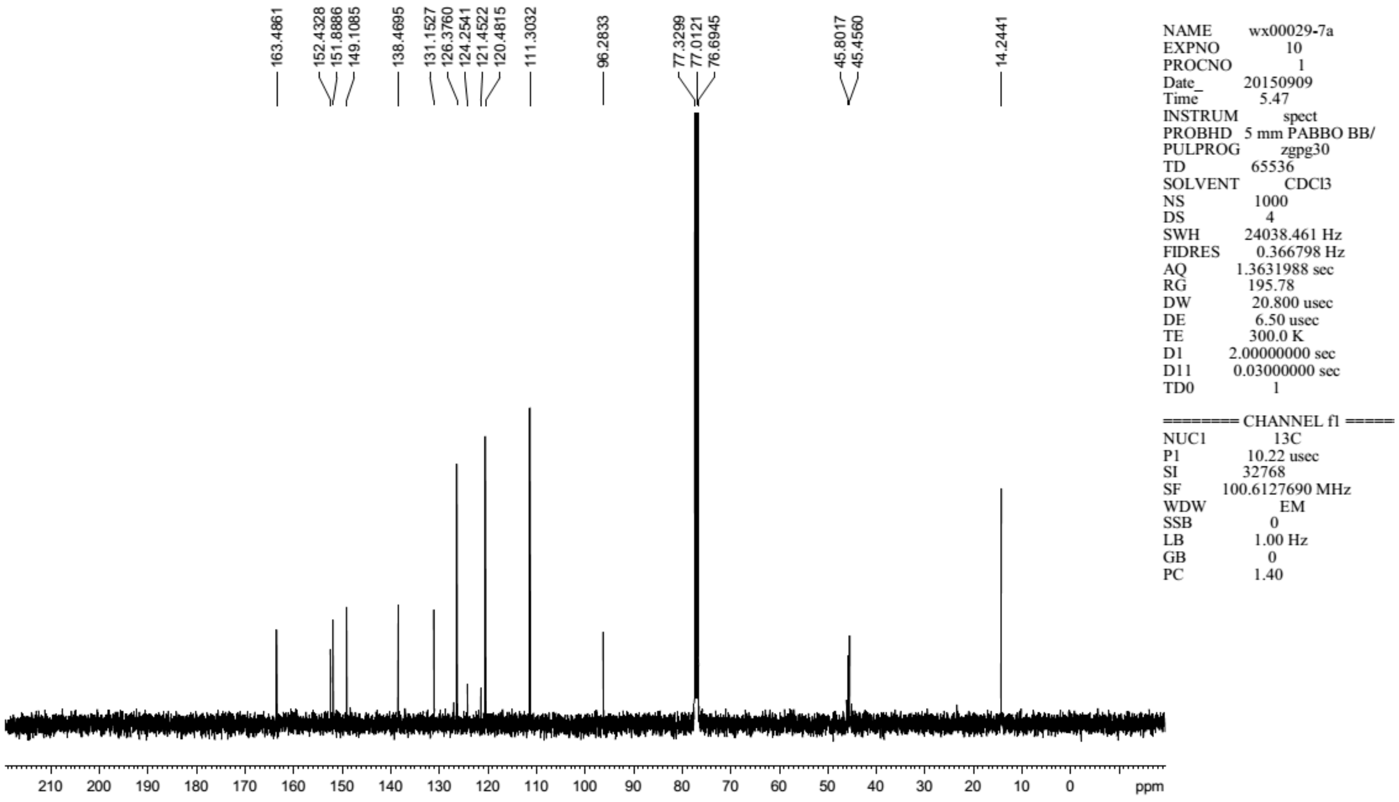

Figure S24. ^{13}C-NMR spectrum of **7a**.

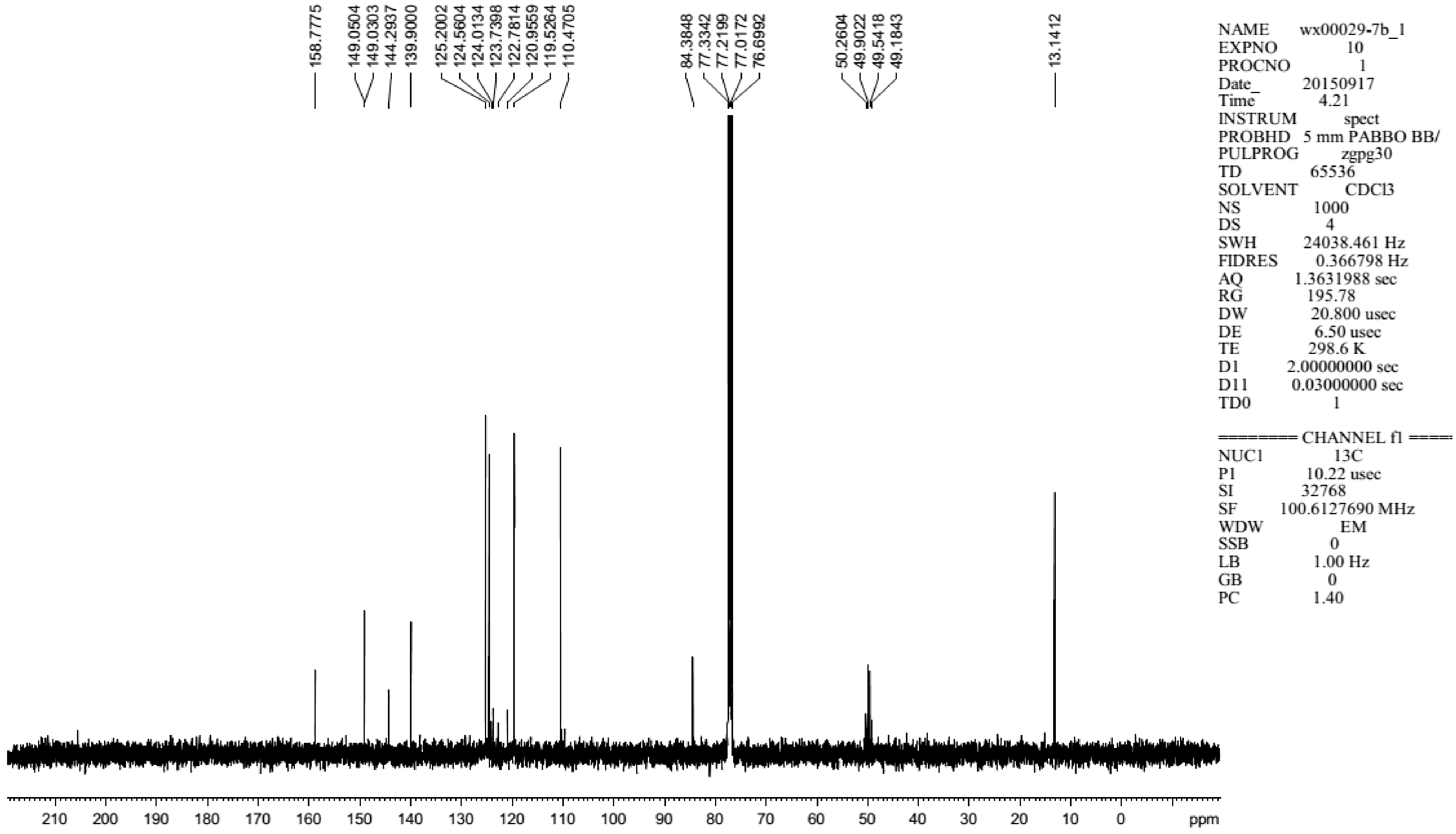

Figure S25. ^{13}C-NMR spectrum of **7b**.

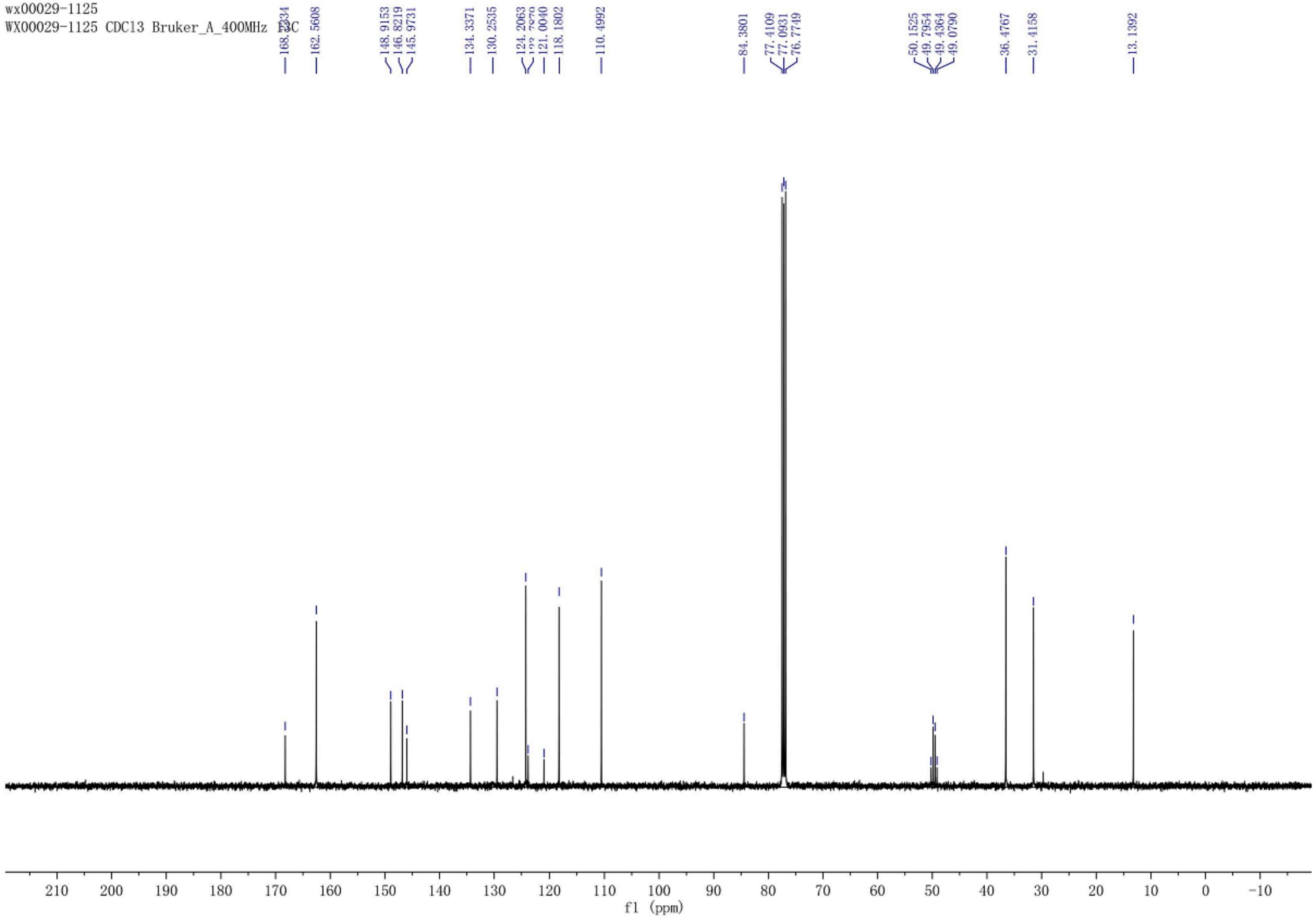

Figure S26. ^{13}C-NMR spectrum of **7c**.

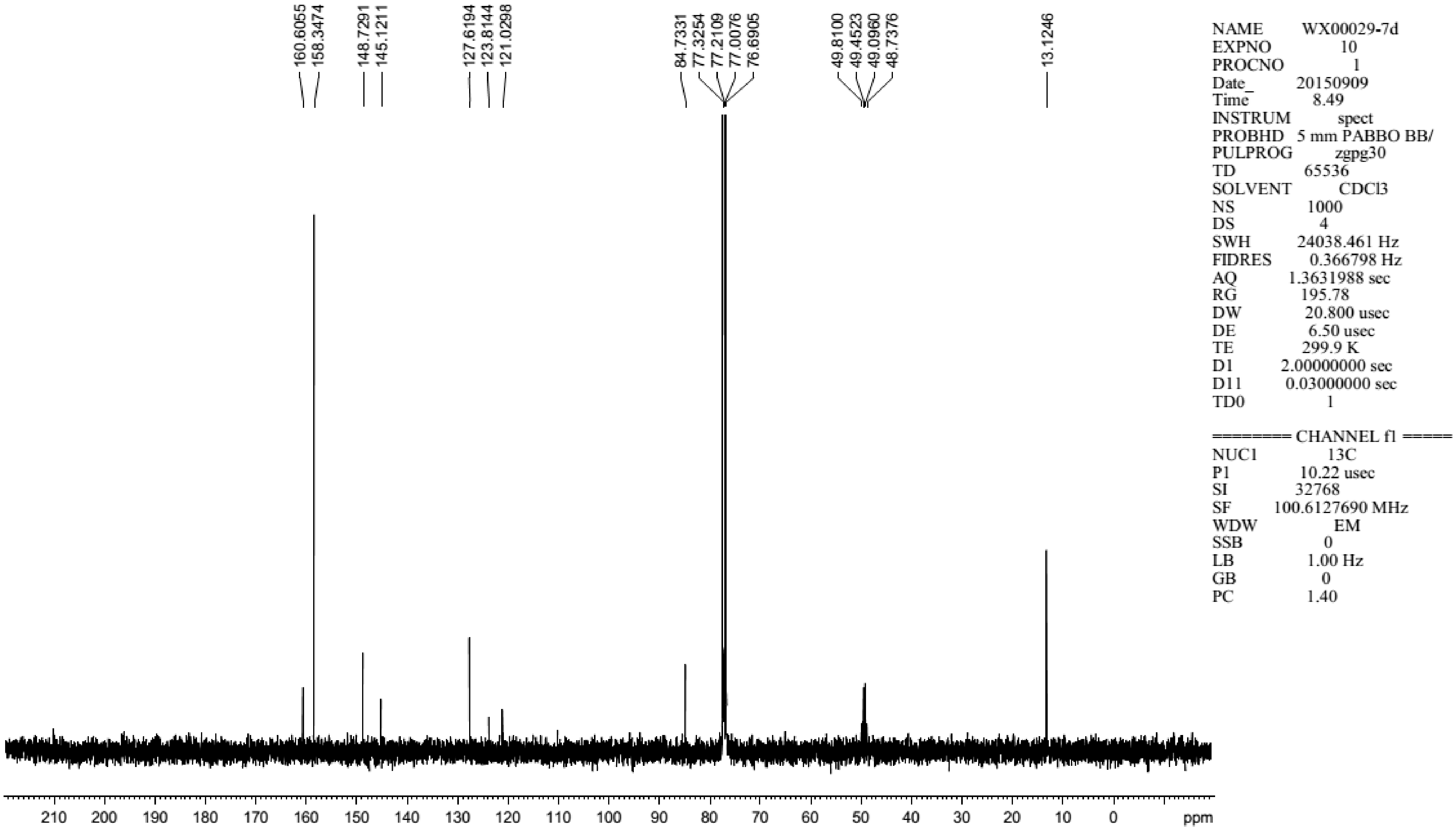

Figure S27. ^{13}C-NMR spectrum of **7d**.

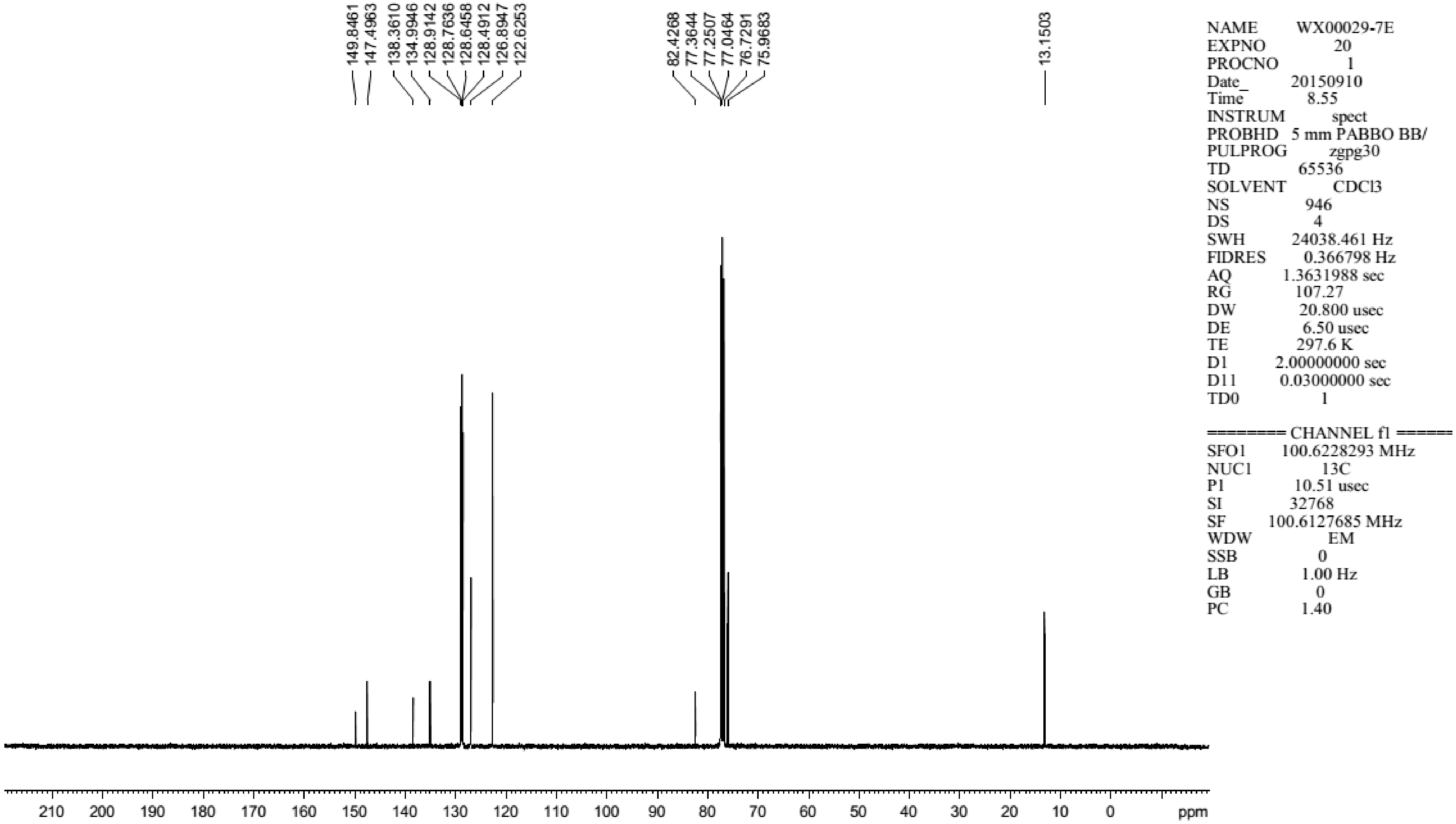

Figure S28. ^{13}C-NMR spectrum of **7e**.
